# Antioxidant Role of Probiotics in Inflammation-Induced Colorectal Cancer

**DOI:** 10.3390/ijms25169026

**Published:** 2024-08-20

**Authors:** Sevag Hamamah, Andrei Lobiuc, Mihai Covasa

**Affiliations:** 1 Department of Basic Medical Sciences, College of Osteopathic Medicine, Western University of Health Sciences, Pomona, CA 91766, USA; sevag.hamamah@westernu.edu; 2 Department of Internal Medicine, Scripps Mercy Hospital, San Diego, CA 92103, USA; 3 Department of Medicine and Biomedical Sciences, College of Medicine and Biological Science, University of Suceava, 7200229 Suceava, Romania; andrei.lobiuc@usm.ro

**Keywords:** gut microbiota, reactive oxygen species, antioxidants, oxidative stress, bile acids

## Abstract

Colorectal cancer (CRC) continues to be a significant contributor to global morbidity and mortality. Emerging evidence indicates that disturbances in gut microbial composition, the formation of reactive oxygen species (ROS), and the resulting inflammation can lead to DNA damage, driving the pathogenesis and progression of CRC. Notably, bacterial metabolites can either protect against or contribute to oxidative stress by modulating the activity of antioxidant enzymes and influencing signaling pathways that govern ROS-induced inflammation. Additionally, microbiota byproducts, when supplemented through probiotics, can affect tumor microenvironments to enhance treatment efficacy and selectively mediate the ROS-induced destruction of CRC cells. This review aims to discuss the mechanisms by which taxonomical shifts in gut microbiota and related metabolites such as short-chain fatty acids, secondary bile acids, and trimethylamine-N-oxide influence ROS concentrations to safeguard or promote the onset of inflammation-mediated CRC. Additionally, we focus on the role of probiotic species in modulating ROS-mediated signaling pathways that influence both oxidative status and inflammation, such as Nrf2-Keap1, NF-κB, and NLRP3 to mitigate carcinogenesis. Overall, a deeper understanding of the role of gut microbiota on oxidative stress may aid in delaying or preventing the onset of CRC and offer new avenues for adjunct, CRC-specific therapeutic interventions such as cancer immunotherapy.

## 1. Introduction

Colorectal cancer (CRC) consistently contributes to global mortality, ranking as the second leading cause of cancer-related deaths and the third most diagnosed cancer worldwide [1]. A recent study by GLOBOCAN, the Global Cancer Observatory, which included data on 36 various cancers from 185 countries, estimates that CRC accounted for 9.6% of new cancer cases and 9.3% of new cancer deaths in 2022 [2]. GLOBOCAN also projects that the yearly incidence of CRC will increase from 1.9 million new cases in 2020 to 3.6 million new cases by 2040, with CRC-related deaths rising from 930,000 to 1.6 million during the same period [3]. Although the majority of CRC diagnoses occur in individuals over the age of 70 [4], the incidence of CRC in those under 50 has been rising in recent years, prompting the American Cancer Society to recommend earlier screening [5]. Due to the global burden of the disease and its high mortality rate, significant research has been directed towards understanding the factors driving CRC pathogenesis and improving preventative measures [6,7]. Currently, the non-modifiable risk factors for CRC development include sex, race, genetics, and the presence of inflammatory bowel disease [7], while the modifiable risk factors include dietary habits, physical inactivity, obesity, and smoking [6]. Emerging evidence has shown that the composition of intestinal bacteria, collectively termed the gut microbiota, can influence or protect against CRC pathogenesis by acting as a key intermediary of these modifiable risk factors [8,9,10]. For example, the introduction of a high-fat diet in animal models has been shown to induce tumorigenesis through significant shifts in microbial composition, including increasing pathogenic and inflammatory microbial species such as *Alistipes* while reducing probiotic bacteria like *Parabacteroides* [11]. Cigarette smoking has a similar, unfavorable effect on gut microbiota, enriching species like *Eggerthella lenta* and depleting *Parabacteroides* and *Lactobacillus* to activate oncogenic signaling in the colonic epithelium [9]. There are multiple proposed mechanisms by which these shifts in microbial composition promote CRC development including, but not limited to, inflammatory signaling [12], direct or indirect DNA damage through the production of harmful metabolites [13,14], histone modification [15], upregulating oncogenic genes [11], inducing tumor resistance to treatment [16], and promoting the formation of reactive oxygen species (ROS) [17].

Of particular interest, sustained increases in ROS play a central role in activating carcinogenic signaling pathways by acting as secondary messengers to induce tumorigenesis and cancer progression [18]. Interestingly, microbiota are critical modulators of ROS and oxygen free radical generation, particularly in states of dysbiosis [19]. As such, it has been demonstrated that commensal bacterial species can suppress inflammatory signaling through antioxidant properties, whereas pathogenic bacterial species exert the opposite effect [20]. Additionally, the gut microbial composition has also been shown to be influenced by states of increased oxidative stress with colorectal cancer-associated bacteria adapting their transcriptomes to defend against microenvironment pressures, leading to the pervasiveness of inflammatory gut microbiota [17]. For example, *Escherichia coli* from cancerous microenvironments with increased oxidative stress displayed greater activation of genes that induce virulence, host colonization, metabolite uptake, and survivability compared to their non-cancerous counterparts [17]. Given this reciprocal influence between the gut microbiota and oxygen free radicals, probiotics have emerged as an adjuvant therapeutic method with antioxidant properties. They can mitigate oxidative stress by augmenting antioxidant and anti-inflammatory signaling pathways, promoting the production of antioxidant microbiota-derived metabolites, and enhancing the activity of antioxidases [21]. To date, studies have strongly supported the role of probiotic administration in inflammatory gut conditions [22,23] and accumulating evidence demonstrated their anti-proliferative and anti-inflammatory efficacy in inflammation-driven CRC [24,25,26,27,28].

In this review, we present emerging evidence demonstrating the mechanisms by which unfavorable taxonomical shifts in the gut microbiota generate sustained increases in reactive oxygen species to initiate inflammatory signaling and resulting carcinogenesis. First, we briefly describe the role of ROS and oxygen free radicals in tumorigenesis as it pertains to CRC. In the process, we identify key gut microbial species and important microbiota-derived metabolites that contribute to ROS formation and modulate oxidative status in CRC models. Lastly, we discuss the role of probiotics in reducing oxidative stress by enhancing antioxidase concentrations and mitigating ROS-mediated inflammatory signaling, thereby aiding in the prevention and treatment of inflammation-driven CRC.

## 2. Reactive Oxygen Species, Oxidative Stress, and Colorectal Cancer (CRC)

Under basal conditions, ROS serve as essential signaling molecules, controlling multiple aspects of cellular physiology including the mediation of growth factors, transcription, and immunomodulation [29]. Endogenously, the majority of ROS are produced via oxidative phosphorylation and the mitochondrial electron transport chain with additional contributions from pro-oxidative enzymes such as nicotinamide adenine dinucleotide phosphate oxidase (NOX), nitric oxide synthase (NOS), xanthine oxidases (XO), cyclooxygenases (COX), and lipoxygenases [30,31]. Exogenous factors such as air pollution, radiation, diet, smoking, and drugs also significantly contribute to the total amount of cellular ROS [32]. When in excess, the accumulation of ROS and resulting imbalance between oxygen free radicals and antioxidants lead to a pathological disruption of normal cellular physiology [33,34]. Specifically, oxidative stress is shown to confer cell damage, DNA mutations, inflammatory stress, and the dysregulation of growth factors, which in combination can lead to tumorigenesis [29].

As it pertains to CRC, studies have elucidated potential mechanisms by which tumorigenesis may occur in direct association with the accumulation of ROS [35,36]. Importantly, ROS-mediated pathways have been shown to induce DNA damage and the subsequent tumorigenic mutations commonly seen in CRC pathology including *p53*, *APC*, and *BRAF* [37]. It is estimated that over 50% of human colorectal tumors are derived from *p53* mutations [38], with the oxidation of DNA bases contributing to the mispairing of guanine and cytosine with adenine and thymine bases [39]. Specifically, the DNA oxidation of guanine and cytosine bases results in the formation of 8-oxoguanine and 5-hydroxycytosine, respectively, leading to mispairing during DNA replication and high mutation rates [35]. Interestingly, it has been reported that 8-oxoguanine concentrations are higher in the urine of CRC patients compared to healthy controls, likely due to the enhanced DNA excision rates of mismatched bases and the subsequent excretion [40]. 

In addition, lipid peroxidation is also a potent inducer of CRC development [36,41]. Byproducts of lipid peroxidation pathways are among the most significantly elevated in colitis-associated CRC [36]. For example, the administration of low doses of epoxyketooctadecenoic acid (EKODE) has been found to exacerbate intestinal barrier dysfunction, lipopolysaccharide, and bacterial translocation along with CRC development via inflammatory signaling pathways such as NF-κB and c-Jun N-terminal kinases (JNK) [36]. Similar results have been observed with other lipid peroxidation byproducts including 4-hydroxynonenal (4-HNE) and 12,13-epoxyocyadecenoic acid (EpOME), both of which augmented inflammation and contributed to colorectal tumorigenesis [42,43]. Proteins are also affected by oxidative stress and confirmational changes in their structure can contribute to CRC development. Sulfur-containing amino acids (cysteine and methionine) are easily oxidized, leading to the formation of undegradable protein aggregates, notably of important enzymes, structural proteins, and receptors [44]. A comparative study identified 31 proteins containing oxidation-sensitive cysteines that are associated with tumorigenesis, with cysteine oxidation found to be correlated with CRC-associated changes [45]. Therefore, dysregulated protein degradation systems are a hallmark of CRC, and targeting these pathways has become of recent interest in cancer treatment [46]. 

Though the accumulation of ROS is shown to confer carcinogenic changes in colorectal cancer cells, researchers have harnessed the apoptotic and senescence-inducing capabilities of ROS as an anti-cancer therapeutic modality [47]. Within their tumor microenvironments, uncontrolled proliferation in cancer cells increases ATP requirements and concurrently upregulates oxidative phosphorylation and ROS concentrations. However, intrinsic antioxidant activation is also upregulated in cancer cells, reducing oxidative stress to levels comparable to non-cancerous counterparts. It is important to note the duality of ROS generation within cancer cells, as moderate ROS levels may induce cancer survival and proliferation, while the overload of ROS induces apoptosis and preferential destruction of tumor cells [48]. Toxic accumulation of ROS leads to endoplasmic reticulum stress, triggering apoptotic pathways, most notably via inositol requiring enzyme-1 (IRE1) signaling to induce autophagy and senescence [49]. Taken together, these findings highlight the versatility of ROS and the importance of redox balance in CRC cell physiology.

### 2.1. Antioxidants, Nrf2-Keap1, and Carcinogenesis

Given the pro-carcinogenic influence of ROS accumulation on CRC pathogenesis, cellular antioxidant defense mechanisms serve an important role in alleviating oxidative stress [50]. A recent large-scale study suggests that higher exposure to antioxidants and corresponding oxidative balance correlates negatively with the onset of CRC [51].

**Figure 1:**
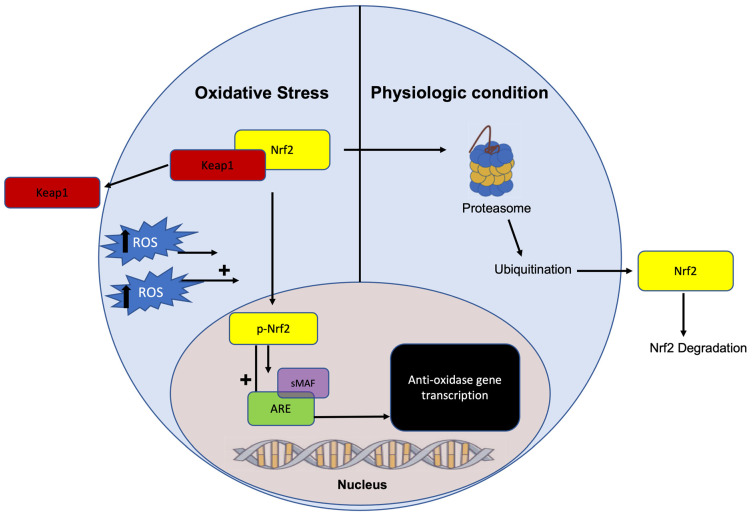
Visualization of the Nrf2-Keap1 pathway and antioxidant enzyme transcription. Under oxidative stress, Nrf2 is phosphorylated and dissociates from Keap1. Phosphorylated Nrf2 then translocates to the nucleus where it binds to small musculoaponeurotic fibrosarcoma (sMAF), activating the antioxidant response element (ARE). This initiates antioxidant enzyme gene transcription including superoxide dismutase, catalase, glutathione peroxidase, glutathione-S-transferase, and heme-oxygenase 1. Conversely, under physiological conditions, Keap1 targets Nrf2 for proteasomal degradation, and antioxidant enzymatic transcription is not upregulated. Abbreviations: Nrf2, nuclear factor erythroid 2; Keap1, Kelch-like ECH-associated protein 1; ROS, reactive oxygen species; p-Nrf2; phosphorylated Nrf2; ARE, antioxidant response element; sMAF, small musculoaponeurotic fibrosarcoma.

To further defend against oxidative and electrophilic stress, eukaryotic cells utilize the Nrf2-Keap1 pathway to activate an antioxidant response element (ARE) and related gene network to promote the production of antioxidant enzymes [52]. In an unstressed oxidative environment, Kelch-like ECH-associated protein 1 (Keap1) binds to and inhibits nuclear factor erythroid2-related factor (Nrf2). Alternatively, states of oxidative stress induce a conformational change in Keap1, releasing Nrf2, which then translocates to the nucleus to activate ARE [52]. The activation of ARE initiates the transcription of antioxidant enzymes such as superoxide dismutase (SOD), catalase (CAT), glutathione peroxidase (GPx), glutathione-S-transferase (GST), and heme-oxygenase 1 (HO-1), which mitigate redox imbalance and free radical damage [53] (Figure 1).

The importance of the Nrf2-keap1 antioxidant pathway in carcinogenesis is demonstrated through studies with Nrf2 knockout mice which exhibit oxidative toxicity and significant inflammation compared to controls [54,55]. More specifically, in an Azoxymethane/Dextran Sulfate Sodium model of colitis-associated CRC, Nrf2 knockout was associated with higher tumor incidence, indicating a critical role of Nrf2-dependent inflammatory suppression in mitigating CRC development [54]. Increased inflammatory and oxidative markers in Nrf2 deficient mice promoted the proliferation of intestinal crypt cells, increasing the risk and presence of mutations leading to carcinogenesis [54]. Similarly, another study with Nrf2 deficient mice showed a higher incidence of tumor cells and identified 23 novel Nrf2-related genes that indicated poorer prognosis in CRC tumor samples [55]. Aging also dysregulates Nrf2 activity, increasing tumorigenesis via ROS-mediated DNA damage and mutations [56], which may also play a role in CRC development and higher prevalence in older generations. Telomeres, the protective caps of eukaryotic chromosomes, are highly susceptible to oxidative damage [57], and in combination with Nrf2 dysregulation and unbalanced redox status, oxidative stress can lead to mutations, genomic instability, and carcinogenesis [56].

Although the Nrf2 pathway is necessary for mitigating oxidative stress to prevent the onset of colorectal carcinogenesis, fully malignant cells exhibit an opposite fate in response to excessive Nrf2 signaling [58]. Specifically, Nrf-2 activity may induce cancer resistance to chemotherapy and enhance tumor growth by protecting against ROS-mediated cancer cell destruction [58,59]. A recent study using alpha-hederin as an anti-CRC therapy observed that lower activation of the Nrf2-Keap1 pathway conferred better treatment outcomes, notably due to its role in modulating tumor microenvironments [60]. Interestingly, the tumor to normal tissue ratio of Nrf2-Keap1 pathway activation has also been linked to lymphovascular invasion in colorectal cancer [61]. It is hypothesized that oncogenic factors promote conformational changes in the Nrf2-Keap1 complex to prevent Nrf2 degradation [62,63]. Therefore, colorectal cancer cells induce the aberrant activation of Nrf2 to reduce oxidative stress within the tumor microenvironment, enhancing their survival [64]. Excess Nrf2 activity worsens chemotherapy resistance and confers poorer prognosis indicating that Nrf2 inhibitors may play an anti-cancer role in this setting [63].

### 2.2. Inflammatory Signaling, Oxidative Stress, and Carcinogenesis

Inflammation is a significant risk factor for CRC development as those with inflammatory bowel disease (IBD) experience up to a three-fold increased incidence of the condition [65]. IBD is a condition that is characterized by chronic relapsing and remitting intestinal inflammation, which is believed to result from persistent immune responses triggered by multiple factors including genetic predisposition and interactions with gut microbial components [66]. The two primary subtypes of IBD are Crohn’s disease (CD) and Ulcerative colitis (UC) [66]. CD is known to cause transmural inflammation, affecting the entire gastrointestinal tract, particularly the terminal ileum, and is marked by skip lesions [67]. In contrast, UC typically begins in the rectum and causes continuous inflammation that extends proximally, almost exclusively affecting the colon [68]. When uncontrolled, both subtypes can lead to carcinogenic progression, with UC having a slightly higher tendency to induce CRC due to localized inflammatory changes to the colon [69].

Recent research has identified oxidative stress-related genetic risk loci associated with the onset of IBD and its subsequent carcinogenic progression [70]. Genome-wide associated studies have revealed polymorphisms within the *NADPH quinone oxidoreductase* (*NQO1*) and *superoxidase dismutase 2* (*SOD2*) genes. *NQO1* polymorphisms have been linked to anti-inflammatory therapeutic resistance, while mutations in *SOD2* correlate with an earlier age of onset in UC patients [71]. Additionally, meta-analyses have shown that *GST M1* null genotype mutations increase susceptibility to IBD in certain populations [72,73], but not in others [74]. Another genetic locus linked to both the susceptibility and progression of UC involves the Nrf2 transcription factor encoded by the *nuclear factor erythroid-derived 2-like 2* (*Nfe2L2*) gene, which can induce inflammation through the uncontrolled production of ROS [75]. Furthermore, the *paraoxonase* (*PON*) genetic loci within chromosome 7 have been associated with IBD development, with a single amino acid mutation (arginine to glutamine) at position 192 showing a statistically significant difference in susceptibility to or protection against the condition [76].

Chronic inflammation in IBD induces carcinogenesis through multiple mechanisms including intestinal epithelial cell dysplasia via *p53* or *APC* pathways initiated by chromosomal instability, microsatellite instability, and hypermethylation [77]. Oxidative stress induces pro-inflammatory signaling pathways like nucleotide-binding domain, leucine-rich containing family 3 (NLRP3), and nuclear factor kappa beta (NF-κB), which constitutively damage the intestinal barrier and indirectly contribute to colorectal carcinogenesis through increasing susceptibility to dysplastic transformation [78,79]. More specifically, the influence of oxidase stress on NLRP3 inflammasome activation stems from increased mitochondrial NADPH oxidase activity and endoplasmic reticulum stress [80,81]. For example, in colitis-induced CRC mice, a high-cholesterol diet increased tumorigenesis through the mitochondrial ROS-mediated upregulation of the NLRP3 inflammasome [82]. Specifically, NLRP3-ASC assembly increased IL-1β concentrations, augmenting chronic inflammation and inducing dysplasia [82]. Interestingly, mitochondrial ROS directly contributes to NLRP3 relocation to endoplasmic structures, initiating interactions with its adaptor molecule, ASC, and activating the inflammasome [83]. In turn, the overactivation of NLRP3 induces colitis through the upregulation of pro-inflammatory cytokines within intestinal epithelial cells, sustained macrophage activity, and recruitment of effector T cells [84]. This strongly influences carcinogenesis as CRC tumors are densely surrounded by macrophages with strong NLRP3 expression which contributes to greater cell migration, invasion, and metastasis, leading to a poorer prognosis [85]. 

Understanding the pro-inflammatory and immunoregulatory role of NF-κB is also crucial in CRC pathogenesis [86]. Oxidative stress modulates NF-κB activity as ROS, notably hydrogen peroxide, and phosphorylates the inhibitor of nuclear factor kappa B (IκB), leading to NF-κB release and translocation to the nucleus [87]. The subsequent activation of NF-κB regulates key components of innate and adaptive immune responses including cytokine transcription and inflammasome activation and is a hallmark of inflammatory bowel conditions when dysregulated [88]. More specifically, the byproducts of NF-κB activation encompass a wide range of cellular processes including but not limited to the induction of pro-inflammatory cytokines such as tumor necrosis factor-α and interleukin-1, the release of chemokines like CXCL12 and CXCL13, the activation of multiple transcription factors involved in angiogenesis and inflammation, the alteration of adhesive molecule expression like cadherins, and the secretion of antimicrobial peptides [89,90]. Interestingly, the expression of NF-κB is found to be comparatively elevated in patients with colorectal cancer when compared with IBD, supporting its role in adenoma to carcinoma transformation [91]. The NF-κB signaling pathway has been associated with oncogenic mutation notably those in the *p53*, *BRAF*, and *APC* genes, which comprise a large percent of colorectal cancers [92]. NF-κB signaling is also implicated in cell cycle progression and dysregulation induces constitutive expression of proliferative genes [93]. Suppressing NF-κB activation has been shown to induce cell cycle arrest at the G0/G1 stage, reducing colorectal cancer growth [94]. NF-κB inhibitors also suppress the IL-1-induced proliferation of CRC cells in inflammatory microenvironments by inhibiting IκB phosphorylation [95]. Therefore, targeting the NF-κB pathway to elicit anti-inflammatory and anti-proliferative effects on inflammation-derived CRC development is warranted [96].

Nrf2-mediated pathways may also disrupt NF-κB activation, helping mitigate the development of inflammation-induced CRC [97]. Specifically, Nrf2 activation in response to oxidative stresses induces HO-1 activity, responsible for degrading heme, a potent oxidant, into iron, biliverdin, and carbon dioxide [98]. Concurrently, degradation into these byproducts, particularly carbon dioxide, negatively influences NF-κB translocation into the nucleus, resulting in the downregulation of the pathway and inflammatory effects [99]. Thus, Nrf2-mediated antioxidant signaling plays a crucial role in mitigating the extent of NF-κB-induced inflammation and related inflammatory damage in the onset of colorectal carcinogenesis.

## 3. Gut Microbiota and Colorectal Cancer (CRC)

It is estimated that trillions (10^13^–10^14^) of commensal microbes reside within the human gastrointestinal tract [100]. This collection of intestinal microbes is termed as the gut microbiota while their total genomic composition is referred to as the gut microbiome [100]. When a healthy or favorable gut microbial composition is maintained, gut microbiota confers a myriad of beneficial effects on host physiology including immunomodulation, energy homeostasis, protection from gut bacterial pathogen overgrowth, and reduced gut intestinal barrier permeability [101,102,103]. Gut microbial composition is influenced by both environmental and genetic factors throughout an individual’s lifetime, with diet, antibiotic use, mode of delivery during birth, geographical location, age, and family genetics playing a significant role [104]. Given the multifactorial influence on the compositional makeup of the gut microbiota, perturbations are common and have been linked to multiple disease states, including obesity, dyslipidemia, type 2 diabetes mellitus, neuropsychiatric disorders, inflammatory bowel disease, and even cancers [105,106,107,108]. Over the last few decades, significant data have linked states of dysbiosis to CRC, with the identification of specific taxonomical shifts and trends in microbiota species that may act as biomarkers of CRC as well as species that are associated with CRC diagnosis and progression [11,109]. These trends include increased relative abundances of *Fusobacterium nucleatum*, *Porphyromonas gingivalis*, *Megamonas funiformis*, *Bacteroides vulgatus*, *Bacteroides stercoris*, *Ruminococcus gnavus*, *Dorea longicatena*, *Escherichia coli*, *Clostridium*, *Atopobium parvulum*, and *Actinomyces odontolyticus* [109,110,111,112,113] with generally decreased abundances of protective species such as *Lacticaseibacillus paracasei*, *Clostridium butyricum*, *Streptococcus thermophilus* and *Faecalibacterium prausnitzii*, *Roseburia intestinalis*, and *Eubacterium rectale* [114,115,116,117].

Of the listed perturbations in microbial composition, the most supported evidence exists for *Fusobacterium nucleatum* [118], which is consistently elevated in the fecal samples of affected patients with early to late stages of CRC [110]. For example, the introduction of *Fusobacterium nucleatum* into mice containing the *APC* gene mutation accelerated tumorigenesis through myeloid cell infiltration and pro-inflammatory signaling in CRC [119]. Mechanistically, *Fusobacterium nucleatum* has been described to exert tumorigenic effects through multiple modalities, including altering the gene expression of microRNAs associated with CRC [120], production of harmful metabolites to disrupt autophagy [121], and activating inflammatory signaling via the NF-κB pathway [122]. In addition to supporting tumorigenesis, *Fusobacterium nucleatum* contributes to tumor metastasis through NF-κB dependent mechanisms including the upregulation of cancer migration genes, methylation of mRNA, and inducing macrophage infiltration into tumor cells [123,124,125]. Similarly, *Fusobacterium nucleatum* concentration is shown to modulate cancer immunotherapy targeting immune checkpoint inhibitors, with a direct correlation between the relative abundance of the bacterium and PD-L1 blockade in mouse models [126]. Recent data have also shown that the bacterium can induce chemoresistance through inhibition of ferroptosis, pyroptosis, and apoptosis [127,128,129], making the species crucial to our understanding of both the pathogenesis and augmentation of therapeutic modalities.

In addition to *Fusobacterium nucleatum,* other microbial species are found to have significant association with various stages of CRC. For example, studies suggest that *Atopobium parvulum and Actinomyces odontolyticus* co-occur and increase in abundance in intramucosal and multiple polypoid adenomas [110]. *Atopobium parvulum*, a bacterium with cysteine desulfarase activity, promotes hydrogen sulfide formation within the gut, which can be toxic to colonocytes [130]. The elevation of *Atopobium parvulum* in early-stage CRC and the related overproduction of hydrogen sulfide also lead to further disruption of the gut microbiome and CRC progression in conjunction with other hydrogen sulfide-producing species such as *Fusobacterium nucleatum* [121,130]. Additionally, the analysis of culprit species in CRC pathogenesis has identified multiple species that may serve as potential biomarkers for CRC development including *Fusobacterium nucleatum*, *Peptostreptococcus anaerobius*, *Bacteroides fragilis*, *Parvimonas micra*, *Xanthomonas perforans* and *Clostridium symbiosum* [112,131]. Similarly, *Bacteroides massiliensis*, *Bifidobacterium pseudocatenulatum*, *Corynebacterium appendicis,* and *Alistipes onderdonkii* have been identified as biomarkers that may help differentiate non-cancerous tissue from CRC tissue [132]. Furthermore, species such as *Porphyromomnas gingivalis* are not only linked to CRC pathogenesis, but also provide insight into patient prognosis [111,133]. *Porphyromomnas gingivalis* induces carcinogenesis through activation of the NLRP3 inflammasome, promoting the development of inflammation-driven CRC [111]. In relation to prognosis, studies show that patients with significantly elevated *Porphyromomnas gingivalis* in fecal samples have decreased cancer-specific survivability [133]. Taken together, there is strong evidence for the intricate interplay between the gut microbiota and CRC pathogenesis, treatment, identification, and prognosis.

### 3.1. Microbiota, Reactive Oxygen Species, and Colorectal Cancer

As previously described, the presence of ROS and oxidative stress creates a pro-tumorigenic intestinal environment serving as an important risk factor for CRC development [134] through direct oxidative damage to the intestinal epithelium [135]. Microbiota serve as important modulators of redox status, both positively and negatively depending on the relative abundances of various species and the intestinal microenvironment [136,137]. The disruption of the intestinal barrier via oxidative damage increases permeability, allowing for the translocation of pathogenic microbiota and metabolites, which may drive inflammation-associated CRC [138]. Additionally, damage to the intestinal barrier increases the susceptibility of intestinal stem cells to genotoxic or environmental mutagens which can contribute to uncontrolled inflammatory bowel states [139].

Certain gut microbiota influence carcinogenesis through their oxidative potential and redox properties, including *Peptostreptococcus anaerobius*, *Enterococcus faecalis,* and *Bacteroides fragilis* [140,141,142]. *Peptostreptococcus anaerobius* modulates the tumor microenvironment by increasing ROS levels to induce intestinal dysplasia through pro-inflammatory pathways [141]. Specifically, cholesterol biosynthesis pathways and cell proliferation are upregulated in response to *Peptostreptococcus anaerobius* metabolite binding of Toll-like Receptor 2 (TLR-2) and Toll-like Receptor 4 (TLR-4), promoting ROS formation [141]. Reactive oxygen species were identified as an intermediate of these processes, as the introduction of antioxidants or knockdown of TLR-2 or TLR-4 ameliorates both cell proliferation and cholesterol biosynthesis [141]. Furthermore, Enterotoxigenic *Bacteroides fragilis* (ETBF) secretes a zinc-dependent metalloprotease toxin, known as *Bacteroides fragilis* toxin (BFT), which is strongly associated with colon epithelial cell proliferation through the induction of intestinal inflammation [143]. Specifically, BFT binds to receptors on the colonic epithelial cell, triggering carcinogenesis-associated changes such as the cleavage of E-cadherin, enhancing *Wingless-related integration site* (*Wnt*) signaling, and the release of pro-inflammatory cytokines [143]. E-cadherin, in particular, is implicated in CRC and other cancers, as it serves as an adhesion molecule on epithelial cells, facilitating cell-to-cell interactions. The loss of its functionality is correlated with the formation of colorectal adenomas [144]. Concurrently, the collection of changes induced by BFT exacerbates colonic barrier permeability, induces DNA damage, and enhances the metastatic potential of CRC [143]. In addition, ETBF drives inflammatory stimuli to induce tumorigenesis through the enzyme spermine oxidase (SMO) [140]. SMO oxidizes spermine to catabolize polyamines with inflammatory stimuli serving as the primary stimulus in its enzymatic activity [145]. Its oxidative byproducts are cytotoxic in excess, yielding significant DNA damage and resulting carcinogenesis including those of breast, gastric, and colorectal origin [146,147,148]. In a murine model, the *Bacteroides fragilis* toxin upregulate SMO to induce DNA damage via its ROS byproducts [140]. Further, given its enzymatic association with carcinogenesis, novel SMO inhibitors have been studied with great efficacy in suppressing cell proliferation and migration [149]. Similarly, *Enterococcus faecalis* is thought to be a driver of CRC development through its oxidative capacity [142]. This bacterium is a well-documented producer of extracellular superoxide and induces chromosomal instability, such as aneuploidy, tetraploidy, and cell cycle arrest, in murine colonic epithelial cells [150]. 

In addition to contributing to the formation of oxidative environments, CRC-associated species also protect themselves against oxidative stress through intrinsic adaptation mechanisms to improve survivability [17,151]. *Fusobacterium nucleatum* is an obligate anaerobe that withstands oxidative stress microenvironments allowing for the sustained survivability, attachment, and invasion of target tissues [151]. Until recently, it was known that the bacterium can cope with excess ROS [152], though the mechanisms by which it can do so had not been elucidated. A recent study has identified a five-gene locus in *Fusobacterium nucleatum*, encoding *methionine sulfoxide reductase* (*MsrAB*), a two-component signal transduction system known as *ModR*, and distinct proteins [151]. The significance of this multigene locus is shown through *ModR*-directed regulation of *MsrAB*, conferring resistance to ROS-mediated destruction and increased virulence pertaining to the adherence or invasion of the bacterium to CRC epithelial cells [151]. In addition to *Fusobacterium nucleatum, Escherichia coli* also adapts to oxidative environments with different regulatory and resistance responses observed in cancerous and non-cancerous bacteria [17]. This is demonstrated through the upregulation of the inherent arginine decarboxylase (AdiA) enzyme of *Escherichia coli* with oxidative stress induction [17]. AdiA is described as an important enzyme in withstanding oxidative pressure in bacterial species to enhance survivability [153]. Taken together, these studies provide strong evidence supporting the influence of gut microbiota trends on oxidative environments seen in CRC and vice versa.

#### Antibiotics, Redox Balance, and Colorectal Cancer

Similar to the taxonomical trends observed in CRC pathogenesis, it is important to briefly discuss the effects of microbiota depletion through antibiotic use and its relationship with carcinogenesis, particularly in the context of redox imbalance. Notably, findings from a large cohort study suggest that early-life antibiotic use is significantly associated with an increased risk for colon cancer development across all ages, particularly in individuals under the age of 50 [154]. Additionally, a recent meta-analysis of six studies concluded that individuals with the highest levels of antibiotic exposure had a 10% higher risk of developing colorectal neoplasia compared to those with the lowest antibiotic exposure [155]. This increased risk is thought to be in part secondary to the antibiotic-mediated selection of pro-carcinogenic, resistant bacterial species as well as stress-resistant cancerous cells that lack effective DNA repair mechanisms [156]. 

In the post-antibiotic period, these resistant bacteria and DNA mutations permeate, as evidenced by studies showing resistance of *Fusobacterium nucleatum* and *Escherichia coli* subtypes to multiple antibiotic classes [157,158]. In addition to allowing for the overgrowth of carcinogenesis-associated gut microbial species, studies in rodent models have shown that antibiotic use can potentiate the effect of unfavorable gut microbiota [159]. For example, various antibiotic classes were shown to increase the abundance of heightened adherent invasive *Escherichia coli* (AIEC) while also promoting its further expansion in chronically infected mice [159]. Importantly, inflammation induced by antibiotic use upregulates reactive nitrogen species leading to the production of oxidized metabolites that provide a fitness advantage to these pathogenic bacteria, facilitating their expansion [159]. However, not all antibiotics contribute to carcinogenic changes. For instance, erythromycin, a macrolide antibiotic with unique anti-inflammatory and antioxidative properties has demonstrated chemopreventative effects [160]. Studies have shown that erythromycin suppresses pro-inflammatory signaling by inhibiting NF-κB activation, which in turn reduces the expression of downstream targets such as interleukin-6 and COX2 in colorectal cancer cell lines [160]. Given that COX is an inducer of oxidative stress [161], the significant decrease in its expression suggests reduced oxidative stress in proximal intestinal polyps, which were also reduced by up to 70.9% compared to controls [160].

While excessive antibiotic use generally tends to initiate carcinogenic changes, targeted antibiotic therapy may have therapeutic value once colorectal carcinogenesis has occurred, particularly when used against pathogenic, CRC-associated microbial species such as *Fusobacterium nucleatum*, ETBF, and AIEC [162,163]. For example, Metronidazole, an antibiotic that provides enteric anaerobic coverage, has proven effective against *Fusobacterium nucleatum* [164]. Studies involving rodent CRC xenografts abundant in *Fusobacterium nucleatum* derived from humans showed that Metronidazole treatment reduced *Fusobacterium* load, cancer cell proliferation, and overall tumor growth [164]. Additionally, studies in murine models have demonstrated the efficacy of Cefoxitin, a second-generation cephalosporin, in eradicating ETBF while reducing colonic adenoma formation and median colon tumor numbers [165]. Notably, ETBL is associated with the development of interleukin 17A (IL-17A) dependent, inflammation-induced tumors that require persistent colonization of this *Bacteroides fragilis* subtype for tumorigenesis [165]. Cefoxitin was found to effectively decrease mucosal IL-17A expression in this study [165]. In summary, this subsection underscores the importance of antibiotic stewardship as broad-spectrum or prolonged antibiotic use can contribute to the overgrowth of pathogenic bacterial species and the subsequent onset of CRC. At the same time, there is growing evidence that once CRC has developed, selected antibiotics targeting these CRC-associated bacteria may play a role in treatment alongside chemotherapeutic agents.

### 3.2. Microbiota Metabolites, Reactive Oxygen Species, and Colorectal Cancer

Many of the beneficial and pathogenic effects exerted by gut microbiota on cancer development, progression, and treatment are mediated by the metabolites they produce [166]. Just as taxonomical shifts in gut microbiota have been observed in the literature, their metabolites also display unique and conserved changes in those affected with CRC [131,167,168]. For example, CRC-associated metabolites include elevated concentrations of branched-chain amino acids (BCAAs), secondary bile acids, polyamines, and Trimethylamine-N-Oxide (TMAO), while short-chain fatty acids (SCFAs) are characteristically decreased in affected patients [131,169,170]. Of particular interest to this review, there is substantial evidence linking SCFA, secondary bile acids, and TMAO to pre-cancerous and cancerous oxidative environments as they pertain to CRC. These microbiota metabolites will be further discussed in the following subsections.

#### 3.2.1. Short Chain Fatty Acids (SCFAs), Reactive Oxygen Species, and Colorectal Cancer

SCFAs are the catabolic end products of dietary fermentation reactions catalyzed by gut microbiota-related enzymes providing numerous benefits to the human host. These include promoting intestinal barrier integrity, energy regulation, anti-inflammatory, and antioxidant effects [171]. Human enzymes cannot metabolize certain dietary fibers and resistant starches; therefore, gut microbiota serve a symbiotic benefit in producing these SCFAs, which consist mostly of acetate, propionate, and butyrate [172]. In states of dysbiosis and systemic disease, the concentrations of SCFA are notably altered, often significantly reduced [173]. This pattern is observed in patients with CRC, with affected patients having lower serum and fecal SCFA concentrations than unaffected individuals [167,174,175]. A meta-analysis study showed that lower fecal concentrations of acetate, propionate, and butyrate are associated with a higher risk of developing CRC [174]. As previously mentioned, butyrate-producing genera including *Faecalibacterium, Eubacterium,* and *Roseburia,* are significantly reduced in CRC patients. Interestingly, CRC-associated bacteria, such as *Fusobacterium nucleatum,* contribute to this imbalance by outcompeting butyrate-producing bacteria, thereby decreasing its concentration [175]. 

**Figure 2:**
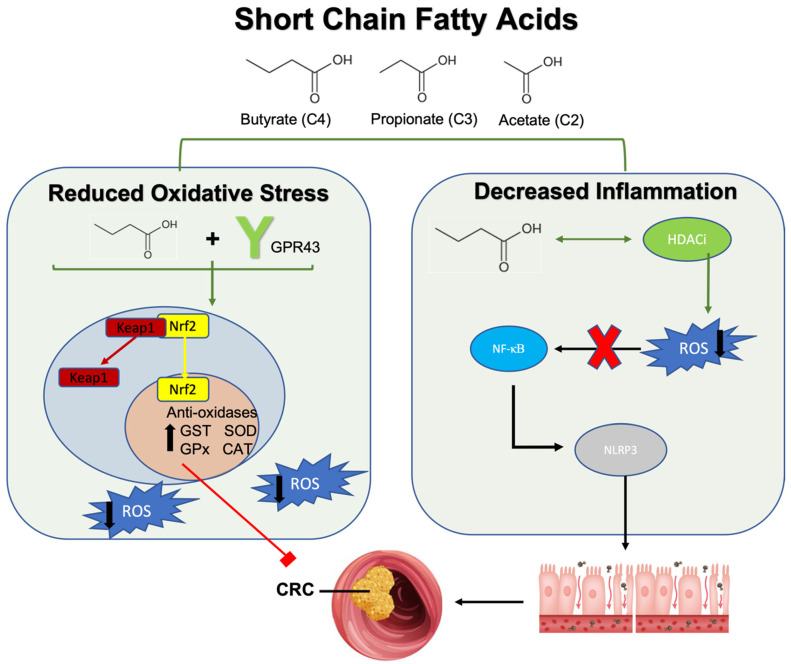
Short chain fatty acids (SCFAs) and antioxidant roles against CRC development. SCFA improves CRC through a reduction in oxidative stress, which enhances antioxidase release and attenuates inflammation. SCFA binding to its receptor, GPR43, and activates the Nrf2-Keap1 pathway. Keap1 dissociates from Nrf2, allowing Nrf2 to enter the nucleus and upregulate the transcription of antioxidant enzymes. This, in turn, reduces ROS concentrations to attenuate the onset of inflammation-induced CRC. On the right, butyrate is shown to enhance HDAC inhibitor activity, which reduces ROS production. Decreased ROS production leads to less activation of the NF-KB signaling pathway and NLRP3 inflammasome, again lessening the development of inflammation-induced CRC. Abbreviations: GPR43, G-coupled Receptor 43; Nrf2, nuclear erythroid factor 2; Keap1, Kelch-like ECH associated protein 1; GST, glutathione-S-transferase; SOD, superoxide dismutase; GPx, glutathione peroxidase; CAT, catalase; ROS, reactive oxygen species; CRC, colorectal cancer; HDACi, histone deacetylase inhibitors; NF-κB, nuclear factor kappa beta; NLRP3, NLR family pyrin domain containing 3.

The mechanisms through which SCFA contribute to the prevention of carcinogenesis and tumor progression are multi-fold, including influencing cell cycle regulation, inflammatory signaling, and cancer signaling pathways as well as suppressing tumor proliferation and metastasis [176]. These metabolic byproducts are known to regulate cancer cell growth through modulating ROS production [175]. Two proposed mechanisms by which SCFA can influence oxidative stress include the activation of its G-coupled protein receptor (GPR41 or GPR43) or its histone deacetylase inhibitor (HDACi) activity [177]. At physiologic levels, butyrate increases the activity of antioxidant enzymes such as glutathione-S-transferase and superoxide dismutase to mitigate oxidative stress [178,179]. SCFA introduction as well as other GPR43 agonists exert similar antioxidant effects and reduce ROS formation [179] (Figure 2). Moreover, GPR43 agonism exerts beneficial effects on inflammatory pathways by improving oxidative stress [180]. For example, in a sepsis-induced inflammatory murine model, the upregulation of the GPR43 gene correlated with reduced ROS-mediated mitochondrial damage while inhibiting NLRP3 inflammasome activity to reduce inflammation, an effect not seen in GPR43 knockout mice [180]. Similarly, GPR43-deficient mice have exacerbated colonic inflammatory responses with elevated pro-inflammatory cytokine concentrations such as TNF-α and IL-17, which is refractory to SCFA treatment [181]. Further, resistant starch diets, rich in SCFA, induced GPR43 mRNA reducing inflammation and decreasing tumor multiplicity and colonic adenocarcinoma formation in a rodent model [182]. Therefore, the SCFA receptor GPR43 plays an important role in reducing oxidative stress and inflammation that could lead to inflammation-driven CRC. 

Histone deacetylase (HDAC) removes acetyl groups on histones, allowing DNA to wrap more tightly, making them more resistant to gene transcription, activation of signaling pathways, and epigenetic modification [183]. Butyrate, the most potent HDACi of the three common SCFAs, assists in inhibiting the removal of acetyl groups [184]. This inherent HDACi capability of SCFA helps suppress the NF-κB signaling pathway [185] and NLRP3 inflammasome activity [186] to protect the intestinal barrier from inflammatory DNA damage induced by ROS production (Figure 1). Importantly, butyrate-mediated histone deacetylation allows for the transcriptional upregulation of antioxidative enzymes such as glutathione-S-transferases (GST) to inhibit the phosphorylation of *extracellular signal-regulated kinase (ERK*) and *mitogen-activation protein kinase* (*MAPK*) signaling in colon cancer cells [187]. *ERK* and *MAPK* overexpression is commonly observed in CRC, as their dysregulation promotes the uncontrolled proliferation of cells [187]. In support of these findings, the accumulation of butyrate has been shown to play an anti-proliferative role by halting cell cycle progression in intestinal epithelial cells, thereby protecting against colonic neoplasia [188]. Research points to histone modification, specifically histone 3 hyperacetylation, as a key factor in the upregulation of cell cycle proteins such as cyclin D1 and p21 [189,190]. Further, through epigenetic modification via HDACi activity, butyrate acts on the Nrf2 promoter to exert potent antioxidant effects [191] (Figure 2). Butyrate administration synergistically promotes Nrf2 accumulation and inhibits histone acetylation through the induction of the AMPK signaling pathway [191]. As such, antioxidant enzymes are upregulated to promote redox balance and attenuate oxidative damage to the intestinal barrier.

However, it should be noted that balanced concentrations of Nrf2 are important, as elevations may also contribute to CRC progression. Significant upregulation of Nrf2 in chemo-resistant cancer cells may make adequate treatment difficult [192,193,194]. Similarly, colonocytes utilize SCFA as a primary energy source under normal circumstances [195]. With cancer being both a genetic and metabolic disease, cancer cells shift their main source of energy to glucose rather than butyrate [196]. As such, inefficiently metabolized butyrate by cancer cells accumulates in the nucleus of cancer-affected cells, functioning as an HDACi [196]. This phenomenon, known as the Warburg effect, works against cancer cells; in the setting of high butyrate, therefore, probiotics that enhance SCFA concentrations, notably butyrate, are beneficial [197]. Further, in CRC cells treated with acetate, propionate, or butyrate, ROS levels become significantly elevated, contributing to cancer cell death and anti-cancer activity [198]. For example, sodium butyrate introduction directly into colorectal cancer cells induced mitochondria-mediated apoptosis and associated ROS generation [199]. It is shown that the ROS production following direct SCFA treatment into colorectal cancer cells also occurs through the reprogramming of metabolic profiles including alterations in macromolecule transport and metabolism, mitochondrial transport, and respiratory chain complex along with elevated ROS production [198]. This excess ROS production further contributes to cancer cell death, making butyrate a potential therapeutic option when directly administered to colorectal cancer cells [200]. Taken together, these studies demonstrate the importance of redox balance and the multifocal contributions of SCFA in modulating oxidative stress as preventative and therapeutic measures in CRC.

#### 3.2.2. Secondary Bile Acids, Reactive Oxygen Species, and Colorectal Cancer

Bile acids are produced in the liver, stored by the gallbladder, and eventually excreted into the upper intestinal tract to facilitate the digestion of lipids and other fat-soluble molecules [201]. Before excretion, primary bile acids are conjugated with taurine and glycine to prevent passive reabsorption in the upper small intestine [202]. Most primary bile acids undergo enterohepatic cycling, where they are reabsorbed in the distal ileum and returned to the liver via the portal blood system [203]. About 5% of primary bile acids reach the colon, where gut microbiota-derived enzymes convert them into secondary bile acids [204]. Bile salt hydrolase (BSH) is a major enzyme in this process liberating glycine and taurine from primary bile acids. BSH-containing bacterial genera include *Clostridium*, *Enterococcus*, *Listeria*, *Bacteroides*, *Bifidobacterium,* and *Lactobacillus* [205]. Similarly, certain gut microbiota have hydroxysteroid dehydrogenase (HSDH) activity which oxidizes hydroxy groups in primary bile acids to diversify the bile acid pool [206]. Interestingly, these reactions, particularly with HSDH, are highly dependent on the redox potential and oxidative status of the microenvironment [207].

**Figure 3:**
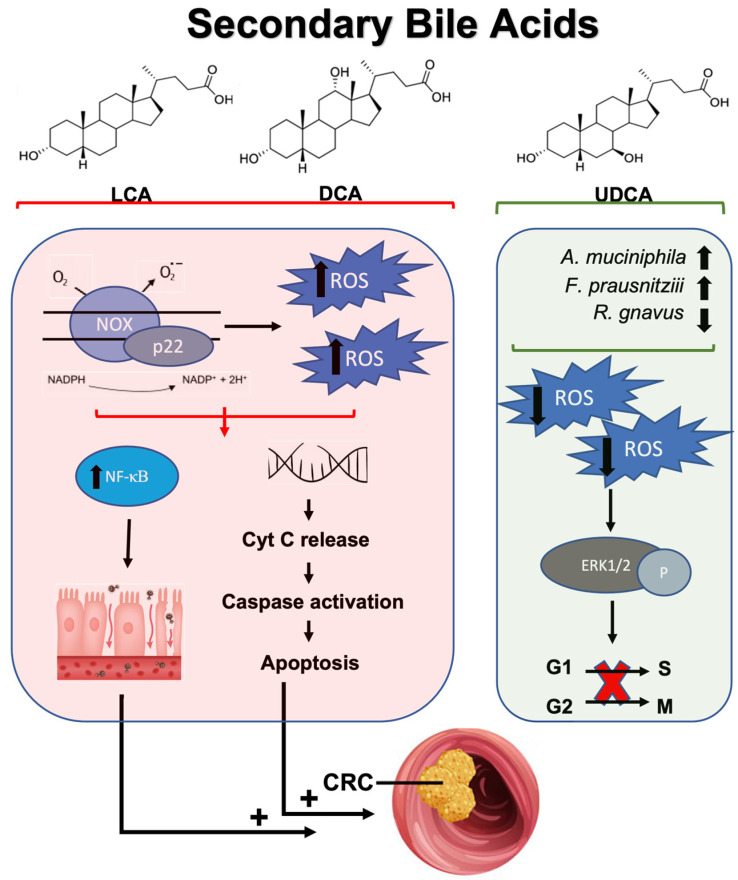
Secondary bile acids and their oxidative role in development or protection against CRC. Lithocholic acid and deoxycholic acid increase oxidative stress through the activation of membrane-bound NADPH oxidases. In turn, this increases NF-κB inflammatory signaling, thereby worsening intestinal tract inflammation. At the same time, ROS production induced from these secondary bile acids can damage DNA, induce cytochrome C release, and initiate apoptosis. These mechanisms influence CRC development. Conversely, ursodeoxycholic acid is a more favorable secondary bile acid that improves microbial composition and decreases ROS production. The stimulation of *ERK1/2* in colon cancer cell lines via UDCA-induced ROS reduction influences cell cycle arrest by preventing cell cycle progression in colon cancer cell lines. This anti-proliferative effect through modulation of oxidative status is beneficial in preventing against progression into CRC. Abbreviations: LCA, lithocholic acid; DCA, deoxycholic acid; UDCA, ursodeoxycholic acid; NOX, NADPH oxidase; ROS, reactive oxygen species; NF-κB, nuclear factor kappa beta; Cyt C, cytochrome C; CRC, colorectal cancer; *ERK1/2, extracellular signal-regulated kinase*.

In colon cancer, secondary bile acids are characteristically elevated and have been associated with pathogenesis and tumor progression through multiple mechanisms, including the modulation of oxidative status within the tumor environment [208,209,210,211]. Secondary bile acids primarily consist of lithocholic acid (LCA) and deoxycholic acid (DCA) with LCA being the most toxic secondary bile acid in relation to CRC, and DCA a close second [212]. LCA and DCA produce ROS through the activation of membrane-bound NADH/NADPH oxidases and phospholipase A2 (PLA2) [213,214]. Excess ROS production can activate cellular signaling pathways, including pro-inflammatory NF-κB, causing sustained inflammation and oxidative DNA damage contributing to CRC pathogenesis [209,211,215]. At the same time, bile acid-induced oxidative stress promotes apoptosis through mitochondrial disruption, resulting in cytochrome C release into the cytosol and activation of caspases [211,216]. However, these cytotoxic effects can affect normal and cancerous cells, with a propensity to have a greater impact on normal cellular physiology. Specifically, these cytotoxic pathways alter the genetic stability of normal colonic cells, while resistant mutant cells may proliferate through the activation of multiple cellular signaling pathways inducing carcinogenesis [213,217]. For example, DCA-mediated ROS production can constitutively activate NF-κB and promote apoptosis resistance in CRC cells [218] (Figure 3).

While most microbial-synthesized secondary bile acids are tumorigenic, the microbial and synthetically produced secondary bile acid, ursodeoxycholic acid (UDCA), may have anti-cancer properties [219,220,221]. Interestingly, UDCA introduction can shift the gut microbial composition towards more favorable bacteria, such as increases in *Faecalibacterium prausnitzii* and *Akkermansia muciniphila,* while decreasing pro-inflammatory *Ruminococcus gnavus* [222,223]. Additionally, UDCA competitively displaces toxic bile acid and can accelerate bile acid enterohepatic circulation [224,225]. Though these favorable effects are well-documented, UDCA naturally comprises about 5% of the total bile acid pool; therefore, the effects of DCA and LCA, particularly in pre-cancerous microenvironments and microbial dysbiosis, may predominate [226]. Further, UDCA inhibits the formation of colon cancer progenitor cells by modulating the oxidative environment [221]. For example, UDCA decreased the total colon cancer cell count without increasing apoptosis. More specifically, UDCA treatment-induced reduction in intracellular ROS to enhance *ERK1/2* phosphorylation in colon cancer cell lines [221]. *ERK1/2* phosphorylation is correlated with cell cycle arrest through G1/S and G2/M transition regulation; therefore, UDCA has anti-proliferative effects through the regulation of cellular oxidative status [221] (Figure 3). These studies highlight the multifactorial influence of secondary bile acids in tumorigenesis and anti-cancer properties, with DCA and LCA having generally harmful effects through ROS production and UCDA having a therapeutic role due to its antioxidant potential.

### 3.3. Trimethylamine-N-Oxide, Oxidative Stress, and Colorectal Cancer

Trimethylamine (TMA) is a byproduct of gut microbiota metabolism of dietary precursors including carnitine, choline, and betaine [227]. TMA-producing bacteria include those that are elevated in CRC such as *Escherichia* and are generally not abundant in individuals with a healthy composition of gut flora [228]. *Escherichia*, specifically, has intrinsic carnitine oxygenase activity, catalyzing the conversion of carnitine to TMA, a reaction dependent on oxygen availability [228,229]. Alternatively, the phylum Firmicutes has choline-TMA lyase and betaine reductase activity which help convert choline and betaine to TMA, respectively [228]. Once produced, TMA is oxidized to Trimethylamine-N-Oxide (TMAO) in the liver by hepatic flavin monooxygenase [230].

**Figure 4:**
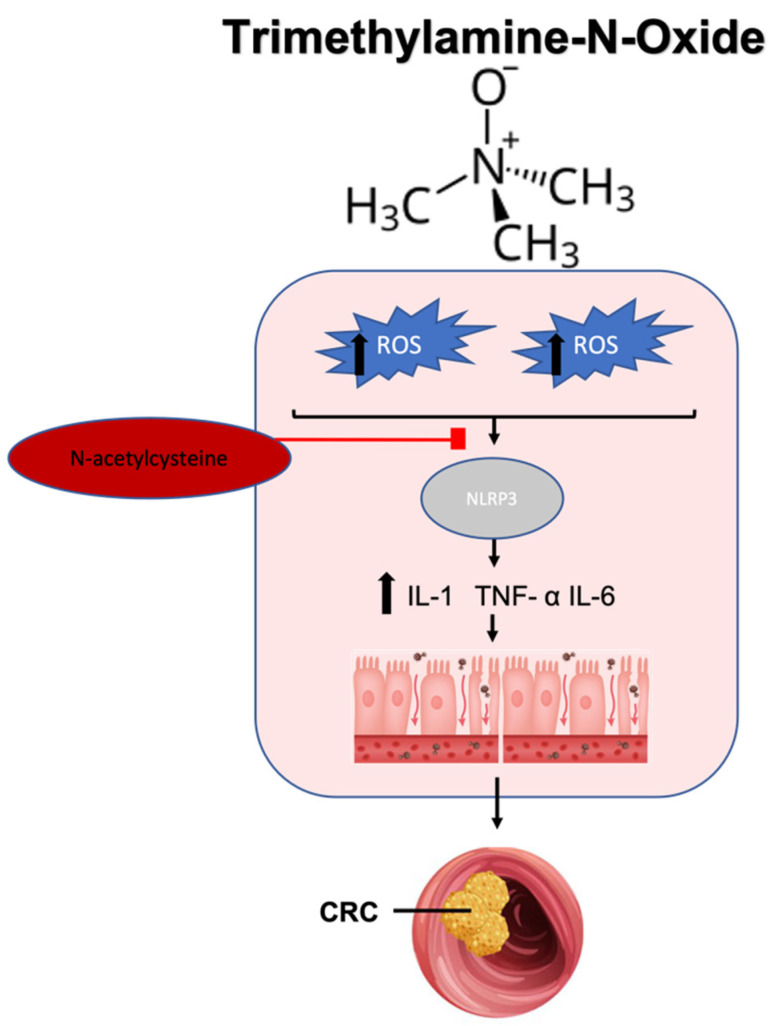
Pro-oxidative role of gut etabolite, Trimethylamine-N-Oxide (TMAO) on CRC development. TMAO increases ROS production to initiate inflammatory signaling, mediating the activation of the NLRP3 inflammasome. NLRP3 activity increases pro-inflammatory cytokines, leading to inflammatory gut pathology leading to the onset of inflammation-induced CRC. The administration of N-acetylcysteine, an ROS inhibitor, attenuates TMAO-induced oxidative stress and related inflammation. Abbreviations: ROS, reactive oxygen species; NLRP3, NLRP3, NLR family pyrin domain containing 3; IL-1, interleukin-1; TNF, tumor necrosis factor; IL-6, Interleukin 6; CRC, colorectal cancer.

TMAO concentrations are elevated in individuals with CRC and have been correlated with carcinogenesis through mechanisms including oxidative stress-driven inflammation, leading to DNA damage [231,232]. Studies have shown that elevated TMAO levels induce oxidative stress, activating the NLRP3 inflammasome pathway to produce pro-inflammatory cytokines in a dose-dependent manner [232]. Another study also noted NLRP3 inflammasome activity following increases in TMAO [233]. Interestingly, treatment with N-acetylcysteine, an ROS inhibitor, reversed these TMAO-mediated effects, further supporting the role of TMAO-induced oxidative stress and related inflammation [232] (Figure 4). Additionally, a recent meta-analysis of 363 manuscripts confirmed associations between TMAO and inflammation in humans, demonstrating sustained and dose-dependent increases in C-reactive protein, an important inflammatory marker [234].

While the influence of TMAO on oxidative stress and inflammation is well-documented, the exact mechanisms conferring CRC pathogenesis require further elucidation. In colon epithelial cells, TMAO induces oxidative stress and apoptosis, inducing NLRP3 activity [235]. However, as mentioned throughout this review, sustained levels of ROS and inflammation are known to confer DNA damage and protein misfolding, leading to carcinogenesis. In other cancers, such as hepatocellular carcinoma and pancreatic cancer, TMAO induces carcinogenesis by upregulating inflammatory signaling pathways and superoxide dismutase activity, promoting inflammation-induced cancers [231,236]. Thus, targeting TMAO has been studied as immunotherapy in treating these cancers [237,238]. TMAO may also serve as a future therapeutic target for inflammation-induced CRC in the context of oxidative stress, though more research is necessary to confirm these findings.

## 4. Probiotics, Antioxidant Properties, and Colorectal Cancer

While harmful gut microbial species have been documented to induce ROS-mediated carcinogenesis, beneficial bacteria with probiotic effects can combat colorectal cancer development and progression through their antioxidant properties [21,239,240]. In conditions of bowel inflammation and elevated ROS, probiotics have been shown to reduce ROS concentrations to non-pathological levels [241]. For example, the daily consumption of a probiotic mixture containing *Lactobacillus acidophilus*, *Lactobacillus rhamnosus,* and *Bifidobacterium bifidum* reduced the mean number of tumors by 40% in an inflammation-induced rodent model of CRC [242]. The influence of probiotic bacteria on preventing carcinogenesis through the modulation of oxidative status is multi-faceted. This includes the microbiota-dependent activation of antioxidative enzymes, regulation of inflammatory signaling pathway, and alteration of circulating microbiota-derived metabolites [243,244,245]. Probiotic microbial genera include first-generation probiotics such as *Bifidobacterium,* and *Lactobacillus* as well as Next-Generation Probiotics (NGPs) like *Akkermansia muciniphila*, *Faecalibacterium prausnitzii*, *Bacteroides fragilis*, *Blautia producta*, and *Clostridium butyricum.* First-generation probiotics are well described to have anti-cancer and anti-inflammatory effects related to CRC, partly by regulating oxidative status through oxidative and inflammatory pathways, such as Nrf2-keap1 and NF-κB signaling, respectively [246,247,248]. The antioxidant role of NGPs in relation to CRC is less extensively studied, but these probiotics have been documented to have beneficial roles in inflammatory bowel states by exerting antioxidant effects [114,249]. Recent studies have clarified the multifaceted impact of NGPs in mitigating oxidative stress to prevent CRC carcinogenesis [114]. For example, *Faecalibacterium prausnitzii* inhibits NF-κB activation to attenuate the proliferation of CRC cell lines, likely due to its strong butyrate-producing capacity [114]. Additionally, treatment with *Faecalibacterium prausnitzii* reduced lipid peroxidation and oxidative stress in an azoxymethane-induced CRC rodent model [114]. Azoxymethane is a toxin that induces CRC by depleting GSH and impairing antioxidant response in colon cells [250]. Aberrant crypt foci, precursors to colorectal polyps, were also reduced in this study, indicating that *Faecalibacterium* may be indicated in CRC prevention.

The following subsections will discuss in detail the mechanisms by which probiotics confer positive changes in combatting oxidative stress and how this influences CRC carcinogenesis and progression, which are also summarized in Table 1. Additionally, probiotics and gut microbiota have also been shown to assist in cancer chemotherapy and immunotherapy [126,237,238,251]. 

### 4.1. Probiotics, Antioxidant Enzymes, and Colorectal Cancer

Probiotics exert beneficial antioxidant effects through the modulation of antioxidant enzymes, such as superoxide dismutase, catalase, glutathione peroxidase, and glutathione-S transferase [21]. Generally, lactic acid bacteria are well documented for having intrinsic oxidative defense enzymes such as NADH oxidase and pyruvate oxidase, which help to maintain redox balance [244,252,253]. For example, a recent study demonstrated that introducing *Lactobacillus bulgaricus* into an animal model increased levels of superoxide dismutase, catalase, and glutathione peroxidase, thereby mitigating oxidative stress [254]. 

Importantly, the positive antioxidant enzymatic changes in probiotics are shown to play a protective role in the onset of CRC-specific tumorigenesis [27,255,256]. Interestingly, the oral administration of *Lactococcus lactis,* a catalase-producing bacterium, prevented 1,2 dimethylhydrazine (DMH)-induced colon cancer [255] (Table 1). This was evidenced by increased catalase activity which led to significantly less ROS-mediated, inflammatory colonic damage and tumor appearance compared to a non-catalase-producing bacterium counterpart in murine models [255]. Further support for these findings comes from studies involving *Lactobacillus plantarum* administered in vitro to DMH-induced colon cancer cells, which similarly increased antioxidant enzyme activity, including SOD, CAT, and GST. This led to a reduction in mean tumor volume and total number of tumors [27]. DMH is a procarcinogen that induces colorectal cancer through a series of metabolic reactions producing ROS that alkylate DNA and induce carcinogenesis [257]. Further, a more recent study found that exopolysaccharides (EPS) derived from the probiotic *Lactobacillus acidophilus* increase the antioxidant enzymes (SOD, CAT, and GPx) concentrations in DMH-induced colon cancer model [256]. EPS-producing bacteria, particularly the *Lactobacillus delbruecki* strain, also reduced lipid peroxidation via increased antioxidant enzyme activity (GSH, SOD, CAT, and GPx), thereby ameliorating inflammatory mucosal damage [258]. EPS from *Lactobacillus* and *Bifidobacterium* have gained attention for their potent anti-inflammatory and antioxidant properties including their inherent antioxidant enzyme capabilities [259,260].

**Table 1:** Effects of probiotics on antioxidant enzymes, antioxidant regulatory pathways, ROS-mediated inflammatory signaling pathways in CRC pathogenesis, and augmentation of current Immunotherapy. Abbreviations: ROS, reactive oxygen species; DMH, 1,2 dimethylhydrazine; SOD, superoxide dismutase; CAT, catalase; GST, glutathione-S-transferase; GPx, glutathione peroxidase; EPS, exopolysaccharides; Nrf2, nuclear factor erythroid related factor 2; Keap1, Kelch-like ECH-associated protein 1; NF-κB, nuclear factor kappa beta; DSS, Dextran Sulfate Sodium; CRC, colorectal cancer; IL, interleukin; CRISPR, clustered regularly interspaced short palindromic repeats; NLRP3; nucleotide-binding domain leucin rich containing family pyrin domain containing 3; PD-1, programmed cell death protein 1.

	Probiotic	Results/Implications	Reference
Antioxidant Enzymes	*Lactococcus lactis*	Increased catalase activity resulting in significantly decreased ROS-mediated inflammatory damage and tumor appearance	[255]
	*Lactobacillus plantarum*	Administration into DMH-induced colon cancer cells, increased antioxidant enzymes (SOD, CAT, and GST) to reduce mean tumor volume and size	[27]
	*Lactobacillus acidophilus*	Produced metabolic derivative, EPS, which increased antioxidant enzymes (SOD, CAT, and GPx) to mitigate DMH-induced colon cancer	[256]
	*Lactobacillus delbruecki*	EPS reduced lipid peroxidation with concurrent increases in antioxidant enzyme activity (SOD, CAT, GPx, and GSH) Ameliorated Inflammatory damage to colonic epithelium	[258]
Nrf2-Keap1	*Lactobacillus casei*	Reduces oxidative and inflammatory stress in enterocytes by activating the Nrf2-Keap1 and NF-KB pathways Nrf2 activation reduced ROS accumulation through the upregulation of GPx	[248]
	*Bifidobacterium bifidum,* *Lactobacillus gasseri*	Activating effects on Nrf2 in combination with vitamin D3 to increase GST and inhibit the onset of colorectal carcinogenesis	[261]
NF-κB	*Faecalibacterium prausnatzii*	Inhibited NF-κB activation to attenuate the proliferation of CRC cell lines Reduced lipid peroxidation and oxidative stress	[114]
	*Lactobacillus fermentum*	Attenuated NF-κB signaling in DSS-induced colorectal cancer Decreased pro-oxidant cyclo-oxygenase 2	[262]
	*Lactiplantibacillus* *plantarum-12*	EPS production alleviated inflammation through inhibition of NF-κB signaling pathway Related reduction in pro-inflammatory cytokines to inhibit inflammation-induced CRC development	[263]
	*Clostridium butyricum*	Decreases the phosphorylation of NF-κB to decrease cytokine activation, ultimately reducing tumor incidence and size in a colitis-induced CRC model Improves microbial composition in the same CRC model	[264]
	*Bifidobacterium longum*	Diminished NF-κB induction in CRC cells to attenuate development of aberrant crypt foci	[265]
VSL#3	*Lactobacillus acidophilus,* *Lactobacillus plantarum,* *Lactobacillus casei,* *Lactobacillus bulgaricus, Bifidobacterium breve, Bifidobacterium longum, Bifidobacterium infantis,* *Streptococcus thermophilus*	Reduced the expression of pro-oxidant enzymes such as cyclo-oxygenase 2 in DSS-induced mice Attenuate IL-6, IL-1β and increased concentrations of regulatory IL-10	[266]
		Attenuated ROS concentrations to reduce pro-inflammatory chemokines and colitis symptoms Reduced barrier permeability	[267]
		Promotes butyrate production, which is found to increase the expression of antioxidants in colitis-associated CRC models	[268]
Immuno-therapy Augmentation	*Lactobacillus rhamnosus GG*	Incorporated as part of a probiotic-CRISPR cancer immunotherapy system that can penetrate hypoxic tumor environments Allowed CRISPR system to reach tumor microenvironment to enhance ROS release and promote cell death	[269]
	*Lactobacillus reuteri*	Metabolite, reuterin, alters tumor microenvironment oxidative status Selectively enhances ROS expression within colorectal cancer cells to suppress tumor growth	[244]
	*Enterococcus faecalis*	Reduced NLRP3 inflammasome activity through attenuation of phagocytosis, which is necessary for activation Prevented the onset of CRC development	[270]
	*Enterococcus durans*	Nanoparticle treatment of probiotic cultures increased folate concentrations ROS-producing capabilities of the treated probiotic decreased CRC viability by 19%	[271]
	*Roseburia intestinalis*	Induction of CD8+ T-cell mediated cytotoxicity through metabolite, butyrate Augmented PD-1 therapy against CRC	[251]
Radiotherapy Augmentation	*Roseburia intestinalis*	Sensitized inflammation-induced CRC cells to radiotherapy treatment through butyrate production Butyrate facilitated autophagy in irradiated cells to induce cell death	[272]

### 4.2. Probiotics, Antioxidant Signaling Pathways, and Colorectal Cancer

As discussed throughout this review, the Nrf-2 antioxidant pathway plays a crucial role in regulating oxidative stress by modulating a broad spectrum of antioxidant enzymes, contributing to the detoxification and elimination of oxygen free radicals [248]. The gut microbiota and their metabolites significantly influence the maintenance of intestinal barrier integrity, partly through the Nrf2-dependent upregulation of epithelial tight junction proteins [273]. Similarly, natural products that improve inflammatory disease and colitis-associated CRC are shown to inhibit inflammatory responses, oxidative stress, and mucosal injury by enhancing gut microbial composition and signaling via the Nrf2/HO-1 pathway. 

The probiotic-mediated activation of Nrf-2 is shown to mediate significant antioxidant and anti-inflammatory effects, thus suppressing the onset of inflammation-induced CRC [248,274]. For example, a recent comparative study on the antioxidant and anti-inflammatory efficacy of probiotic administration in colitis-induced mice showed the down-regulation of Nrf2 and NF-κB related genes and alleviated colitis [274]. Interestingly, the probiotic, containing 88 strains of *Lactobacillus* and *Bifidobacterium spp.,* improved antioxidant enzymatic activity (SOD, CAT, GPX, and GSH), while reducing the concentrations of pro-inflammatory cytokines such as TNF-α [274]. *Lactobacillus casei,* a lactic acid-producing bacterium, has demonstrated a redox role against oxidative and inflammatory stress in enterocytes through activation of both Nrf-2 and NF-κB signaling [248]. In this study, Nrf2 activation by *Lactobacillus casei* reduced ROS accumulation in enterocytes by upregulating glutathione peroxidase [248]. Similarly, *Bifidobacterium bifidum* and *Lactobacillus gasseri* supplementation in conjunction with vitamin D3 also activated Nrf2, with concomitant increases in GST to inhibit the onset of colorectal carcinogenesis in a rodent model [261]. Overall, *Lactobacillus* spp. and their metabolites are well documented to confer potent antioxidant effects through activation of Nrf2 mediated pathways throughout the body, providing further insight into the role of these bacteria in redox balance and treatment of inflammatory disease [275,276,277]. These findings suggest that probiotic supplementation can reduce ROS-mediated damage and inflammatory stress to enterocytes helping delay or prevent the onset of CRC [248,261]. However, it is important to note that the effects of probiotics on Nrf2-keap1 activity in malignant cells are not well studied and the highlighted studies mainly focus on the preventative effects of probiotics on inflammation-induced CRC. 

### 4.3. Probiotics, ROS-Mediated Inflammatory Signaling Pathways, and Colorectal Cancer

Probiotics have significant positive implications in preventing inflammation-induced CRC through mediating ROS-mediated, inflammatory, and cancer-related signaling pathways such as NF-κB [278]. Enhanced oxidative stress can initiate constitutive signaling through the NF-κB pathway, promoting inflammatory damage and related carcinogenesis in the colon [86,279]. Lactic acid bacteria are implicated in mitigating the NF-κB signaling pathway and protecting against carcinogenesis [262,263]. For example, *Lactobacillus fermentum* supplementation in Dextran Sulfate Sodium (DSS)-induced colorectal cancer rodent models attenuated NF-κB signaling pathway signaling by decreasing key proteins IκBα and p65 and target protein cyclooxygenase-2, a known pro-oxidant [262]. DSS induces CRC by causing colonic epithelial inflammatory damage [280], suggesting that probiotics can mitigate inflammatory signaling pathways to delay carcinogenesis [262]. Similarly, *Lactiplantibacillus plantarum-12*-derived EPS significantly alleviated inflammation by inhibiting the NF-κB signaling pathway and reducing pro-inflammatory cytokines in a murine model of inflammation-induced colon cancer [263]. This bacterium also enhances gut microbial composition by promoting the survival of beneficial gut microbiota and reducing the relative contributions of inflammatory species [263]. Additionally, the butyrate-producing *Clostridium butyricum* decreases the phosphorylation of NF-κB and improves microbial composition in a colitis-associated colon cancer rodent model [264]. Inhibition of NF-κB signaling was a catalyst for the observed decreased cytokine activity, increased apoptosis, and reduced tumor incidence and size. Similarly, *Bifidobacterium longum* reduced NF-κB signaling in CRC cells while attenuating the development of aberrant crypt foci in this murine model [265]. Taken together, these studies provide strong evidence linking the NF-κB pathway, a byproduct of increased oxidative stress, to CRC pathogenesis, with probiotics serving as an efficient therapeutic intervention to attenuate signaling.

Further, VSL#3, a probiotic mixture consisting of microbial species from three bacterial genera (*Lactobacillus*, *Bifidobacterium*, and *Streptococcus*) mitigates inflammation-induced colorectal adenocarcinoma development through the modulation of ROS and inflammatory markers [266,267,281]. Specifically, colonic inflammation scores and incidence of colonic dysplastic lesions were significantly reduced following administration, with concurrent reductions in IL-6, IL-1β, and increased concentrations of regulatory IL-10 [266]. Similarly, the expression of pro-oxidative enzymes such as cyclooxygenase-2 was reduced in these DSS-induced mice, corresponding with decreased ROS activity [266]. Follow-up studies also supported the anti-inflammatory role of VSL#3 through observed suppression of IL-6/STAT3 promoting preventative effects on CRC development [281]. The anti-inflammatory effect of this probiotic mixture is elucidated through its influence on enhancing intestinal barrier function in MUC2-deficient mice [267]. MUC2 serves multiple important roles in maintaining intestinal barrier integrity, with the glycoprotein serving as a protective mucin layer and an important food source for beneficial bacteria [282]. In MUC2-deficient mice, VSL#3 alone reduced barrier permeability, while attenuating pro-inflammatory chemokines and colitis symptoms, partly through the attenuation of ROS [267]. Interestingly, these effects were mediated by the gut metabolite, acetate. However, butyrate production from the introduction of VSL#3 has also demonstrated increased expression of antioxidant enzymes in a colitis-associated CRC model [268]. Taken together, VSL#3 and other probiotic formulations play integral roles in inflammatory pathways, often involving mediation of oxidative stress and ROS, to mitigate the progression or development of inflammation-induced CRC.

### 4.4. Probiotics, Antioxidants and Colorectal Cancer Immunotherapy, and Treatment

There is compelling evidence showing that probiotics protect against CRC pathogenesis and progression, in part, by modulating oxidative stress and related inflammatory processes. In recent years, it has been shown that probiotics can influence oxidative microenvironments to enhance cancer immunotherapy in the treatment of colorectal cancer [269,283]. For example, in antibiotic-treated rodent models, cancer immunotherapy via CpG oligonucleotide treatment diminished both cytokine production and ROS-mediated cytotoxicity in cancer-specific cells, indicating poorer therapeutic responses [283]. Further, recent studies have elucidated a probiotic-CRISPR cancer immunotherapy system consisting of *Lactobacillus rhamnosus GG*. This *Lactobacillus* spp. acts as a vector that can penetrate hypoxic tumor environments allowing CRISPR-derived cancer immunotherapy to reach the tumor microenvironment, enhance ROS release, and induce cell death [269]. Additionally, *Lactobacillus reuteri,* a commonly used probiotic, has been shown to reduce the survivability and proliferation of CRC cells by alternating the oxidative status of the tumor microenvironment [244]. Interestingly, its metabolite, reuterin, mediates these positive effects through protein oxidation, selectively enhancing ROS expression within tumor cells to suppress cancer growth [244]. The observed dose-dependent effects of reuterin were observed in colon cancer cells, but not in reuterin-resistant counterpart cells.

*Enterococcus spp.* have also emerged as probiotics with anti-neoplastic properties [270]. While *Enterococcus faecalis* was previously implicated in pro-carcinogenic effects due to colonic epithelial DNA damage via extracellular superoxide and hydrogen peroxide production [284], recent studies have shown the opposite. Heat-killed *Enterococcus faecalis* treatment has been shown to mitigate intestinal inflammation and prevent inflammation-induced CRC [270], by reducing NLRP3 inflammasome activity. This effect was not observed in NLRP3 knockout mice [270], highlighting the importance of the inflammasome in the efficacy of *Enterococcus faecalis* on CRC treatment. Further, a recent study aimed to harness the ROS-producing capabilities to selectively destroy colorectal cancer cells through nanoparticle therapy [271]. Nanoparticle treatment of *Enterococcus durans* cultures increased extracellular folate concentrations, interacting with metabolic pathways involving amino acids, SCFAs, and energy metabolites [271]. The subsequent introduction of this nanoparticle-treated *Enterococcus durans* cell line into HCT 116 colorectal cancer cell lines decreased cell viability by 19%, indicating a novel anti-neoplastic role of the bacterium.

In the inflammation-induced CRC model, recent findings have shown that *Roseburia intestinalis* enhances immunotherapy, specifically anti-PD-1 therapy in CRC via butyrate production [251]. CRC patients exhibited significantly reduced *Roseburia* concentrations, while supplementation induced CD8+ T-cell mediated cytotoxicity through butyrate binding to Toll-like receptor 5 and activation of NF-κB signaling [251]. Similarly, *Roseburia intestinalis* has also been shown to sensitize inflammation-induced CRC to radiotherapy treatment, again through the production of butyrate [272]. In this rodent model, butyrate facilitated autophagy in irradiated cells, inducing cell death [272]. These findings suggest that *Roseburia intestinalis* may play an important role in augmenting immunotherapy through the modulation of oxidative and related immunological factors.

## 5. Conclusions and Perspectives

There is substantial evidence linking the gut microbiota to oxidative status in CRC models. The intricate interplay between microbiota metabolites, ROS production, and tumorigenesis provides valuable insight into colorectal carcinogenesis. ROS play a dual role in CRC, acting as signaling molecules under normal conditions but contributing to tumorigenesis when in excess. ROS can induce DNA damage, lipid peroxidation, and protein modifications, leading to mutations and carcinogenesis. Oxidative stress also promotes inflammatory signaling pathways, such as NF-κB and NLRP3, further contributing to CRC development. The gut microbiota significantly impacts oxidative status and CRC pathogenesis. Certain bacterial species, such as *Fusobacterium nucleatum* and *Escherichia coli*, can enhance ROS production and inflammatory signaling, promoting tumor growth and metastasis. Conversely, beneficial bacteria like *Faecalibacterium prausnitzii* and *Lactobacillus* spp. exhibit antioxidant properties, mitigating ROS-mediated damage and reducing inflammation. Probiotics have shown promise in preventing and treating inflammation-induced CRC. They can modulate oxidative stress, enhance antioxidant enzyme activity, and influence inflammatory pathways. Specific probiotics, such as *Lactobacillus* and *Bifidobacterium*, have demonstrated efficacy in reducing tumor incidence, enhancing immune responses, and improving the efficacy of cancer immunotherapy. The findings reviewed underscore the complex interplay between gut microbiota, ROS, and CRC. Future research should focus on (i) elucidating the precise mechanisms by which specific gut bacteria influence ROS production and inflammatory pathways; (ii) investigating the role of probiotics in different stages of CRC and their potential in combination therapies with the existing treatments; (iii) exploring the impact of diet and lifestyle modifications on gut microbiota composition and CRC risk; (iv) developing targeted probiotic therapies to enhance the antioxidant and anti-inflammatory responses in CRC patients. Incorporating probiotics and other microbiota-targeted therapies into clinical practice could offer a novel approach to CRC prevention and treatment, potentially improving patient outcomes and reducing the global burden of this disease.

## References

[1] Patel S.G., Karlitz J.J., Yen T., Lieu C.H., Boland C.R. (2022). The rising tide of early-onset colorectal cancer: A comprehensive review of epidemiology, clinical features, biology, risk factors, prevention, and early detection. Lancet Gastroenterol. Hepatol..

[2] Bray F., Laversanne M., Sung H., Ferlay J., Siegel R.L., Soerjomataram I., Jemal A. (2024). Global cancer statistics 2022: GLOBOCAN estimates of incidence and mortality worldwide for 36 cancers in 185 countries. CA Cancer J. Clin..

[3] Morgan E., Arnold M., Gini A., Lorenzoni V., Cabasag C.J., Laversanne M., Vignat J., Ferlay J., Murphy N., Bray F. (2023). Global burden of colorectal cancer in 2020 and 2040: Incidence and mortality estimates from GLOBOCAN. Gut.

[4] Murphy C.C., Zaki T.A. (2024). Changing epidemiology of colorectal cancer—Birth cohort effects and emerging risk factors. Nat. Rev. Gastroenterol. Hepatol..

[5] Siegel R.L., Wagle N.S., Cercek A., Smith R.A., Jemal A. (2023). Colorectal cancer statistics, 2023. CA Cancer J. Clin..

[6] Keum N., Giovannucci E. (2019). Global burden of colorectal cancer: Emerging trends, risk factors and prevention strategies. Nat. Rev. Gastroenterol. Hepatol..

[7] Gausman V., Dornblaser D., Anand S., Hayes R.B., O’Connell K., Du M., Liang P.S. (2020). Risk Factors Associated With Early-Onset Colorectal Cancer. Clin. Gastroenterol. Hepatol..

[8] Feizi H., Rezaee M.A., Ghotaslou R., Sadrkabir M., Jadidi-Niaragh F., Gholizadeh P., Taghizadeh S., Ghanbarov K., Yousefi M., Kafil H.S. (2023). Gut Microbiota and Colorectal Cancer Risk Factors. Curr. Pharm. Biotechnol..

[9] Bai X., Wei H., Liu W., Coker O.O., Gou H., Liu C., Zhao L., Li C., Zhou Y., Wang G. (2022). Cigarette smoke promotes colorectal cancer through modulation of gut microbiota and related metabolites. Gut.

[10] Song M., Chan A.T., Sun J. (2020). Influence of the Gut Microbiome, Diet, and Environment on Risk of Colorectal Cancer. Gastroenterology.

[11] Yang J., Wei H., Zhou Y., Szeto C.H., Li C., Lin Y., Coker O.O., Lau H.C.H., Chan A.W.H., Sung J.J.Y. (2022). High-Fat Diet Promotes Colorectal Tumorigenesis Through Modulating Gut Microbiota and Metabolites. Gastroenterology.

[12] Xing C., Wang M., Ajibade A.A., Tan P., Fu C., Chen L., Zhu M., Hao Z.Z., Chu J., Yu X. (2021). Microbiota regulate innate immune signaling and protective immunity against cancer. Cell Host Microbe.

[13] Arthur J.C., Perez-Chanona E., Mühlbauer M., Tomkovich S., Uronis J.M., Fan T.J., Campbell B.J., Abujamel T., Dogan B., Rogers A.B. (2012). Intestinal inflammation targets cancer-inducing activity of the microbiota. Science.

[14] Cao Y., Oh J., Xue M., Huh W.J., Wang J., Gonzalez-Hernandez J.A., Rice T.A., Martin A.L., Song D., Crawford J.M. (2022). Commensal microbiota from patients with inflammatory bowel disease produce genotoxic metabolites. Science.

[15] Singh V., Shirbhate E., Kore R., Vishwakarma S., Parveen S., Veerasamy R., Tiwari A.K., Rajak H. (2024). Microbial Metabolites-Induced Epigenetic Modifications for Inhibition of Colorectal Cancer: Current Status and Future Perspectives. Mini Rev. Med. Chem..

[16] Jiang S.S., Xie Y.L., Xiao X.Y., Kang Z.R., Lin X.L., Zhang L., Li C.S., Qian Y., Xu P.P., Leng X.X. (2023). Fusobacterium nucleatum-derived succinic acid induces tumor resistance to immunotherapy in colorectal cancer. Cell Host Microbe.

[17] Lamaudière M.T.F., Arasaradnam R., Weedall G.D., Morozov I.Y. (2023). The Colorectal Cancer Microbiota Alter Their Transcriptome To Adapt to the Acidity, Reactive Oxygen Species, and Metabolite Availability of Gut Microenvironments. mSphere.

[18] Sarmiento-Salinas F.L., Perez-Gonzalez A., Acosta-Casique A., Ix-Ballote A., Diaz A., Treviño S., Rosas-Murrieta N.H., Millán-Perez-Peña L., Maycotte P. (2021). Reactive oxygen species: Role in carcinogenesis, cancer cell signaling and tumor progression. Life Sci..

[19] Lee K.I., Jo Y., Yuk H.J., Kim S.Y., Kim H., Kim H.J., Hwang S.K., Park K.S. (2024). Potential Phytotherapy of DSS-Induced Colitis: Ameliorating Reactive Oxygen Species-Mediated Necroptosis and Gut Dysbiosis with a New Crataegus pinnatifida Bunge Variety-Daehong. Antioxidants.

[20] Costa G.T., Vasconcelos Q., Aragão G.F. (2022). Fructooligosaccharides on inflammation, immunomodulation, oxidative stress, and gut immune response: A systematic review. Nutr. Rev..

[21] Wang Y., Wu Y., Wang Y., Xu H., Mei X., Yu D., Wang Y., Li W. (2017). Antioxidant Properties of Probiotic Bacteria. Nutrients.

[22] Khalil N.A., NA A.L., JZ A.L., Mohamed Ahmed I.A. (2024). Anti-inflammatory effects of bay laurel (*Laurus nobilis* L.) towards the gut microbiome in dextran sodium sulfate induced colitis animal models. Food Sci. Nutr..

[23] Yu F., Hu X., Ren H., Wang X., Shi R., Guo J., Chang J., Zhou X., Jin Y., Li Y. (2024). Protective effect of synbiotic combination of Lactobacillus plantarum SC-5 and olive oil extract tyrosol in a murine model of ulcerative colitis. J. Transl. Med..

[24] Tan L.T., Chan K.G., Pusparajah P., Yin W.F., Khan T.M., Lee L.H., Goh B.H. (2019). Mangrove derived Streptomyces sp. MUM265 as a potential source of antioxidant and anticolon-cancer agents. BMC Microbiol..

[25] Torres-Maravilla E., Boucard A.S., Al Azzaz J., Gontier S., Kulakauskas S., Langella P., Bermúdez-Humarán L.G. (2023). Assessment of the safety of Levilactobacillus brevis CNCM I-5321, a probiotic candidate strain isolated from pulque with anti-proliferative activities. Benef. Microbes.

[26] Oh N.S., Joung J.Y., Lee J.Y., Kim Y.J., Kim Y., Kim S.H. (2020). A synbiotic combination of *Lactobacillus gasseri* 505 and *Cudrania tricuspidata* leaf extract prevents hepatic toxicity induced by colorectal cancer in mice. J. Dairy. Sci..

[27] Kumar R.S., Kanmani P., Yuvaraj N., Paari K.A., Pattukumar V., Thirunavukkarasu C., Arul V. (2012). Lactobacillus plantarum AS1 isolated from south Indian fermented food Kallappam suppress 1,2-dimethyl hydrazine (DMH)-induced colorectal cancer in male Wistar rats. Appl. Biochem. Biotechnol..

[28] Desrouillères K., Millette M., Bagheri L., Maherani B., Jamshidian M., Lacroix M. (2020). The synergistic effect of cell wall extracted from probiotic biomass containing Lactobacillus acidophilus CL1285, *L. casei* LBC80R, and *L. rhamnosus* CLR2 on the anticancer activity of cranberry juice-HPLC fractions. J. Food Biochem..

[29] Bardaweel S.K., Gul M., Alzweiri M., Ishaqat A., HA A.L., Bashatwah R.M. (2018). Reactive Oxygen Species: The Dual Role in Physiological and Pathological Conditions of the Human Body. Eurasian J. Med..

[30] Zińczuk J., Maciejczyk M., Zaręba K., Pryczynicz A., Dymicka-Piekarska V., Kamińska J., Koper-Lenkiewicz O., Matowicka-Karna J., Kędra B., Zalewska A. (2020). Pro-Oxidant Enzymes, Redox Balance and Oxidative Damage to Proteins, Lipids and DNA in Colorectal Cancer Tissue. Is Oxidative Stress Dependent on Tumour Budding and Inflammatory Infiltration?. Cancers.

[31] Fiaschi T., Chiarugi P. (2012). Oxidative stress, tumor microenvironment, and metabolic reprogramming: A diabolic liaison. Int. J. Cell Biol..

[32] Bhattacharyya A., Chattopadhyay R., Mitra S., Crowe S.E. (2014). Oxidative stress: An essential factor in the pathogenesis of gastrointestinal mucosal diseases. Physiol. Rev..

[33] Pizzino G., Irrera N., Cucinotta M., Pallio G., Mannino F., Arcoraci V., Squadrito F., Altavilla D., Bitto A. (2017). Oxidative Stress: Harms and Benefits for Human Health. Oxid. Med. Cell. Longev..

[34] Carini F., Mazzola M., Rappa F., Jurjus A., Geagea A.G., Al Kattar S., Bou-Assi T., Jurjus R., Damiani P., Leone A. (2017). Colorectal Carcinogenesis: Role of Oxidative Stress and Antioxidants. Anticancer. Res..

[35] Hahm J.Y., Park J., Jang E.S., Chi S.W. (2022). 8-Oxoguanine: From oxidative damage to epigenetic and epitranscriptional modification. Exp. Mol. Med..

[36] Lei L., Yang J., Zhang J., Zhang G. (2021). The lipid peroxidation product EKODE exacerbates colonic inflammation and colon tumorigenesis. Redox Biol..

[37] Lee J.H., Hwang I., Kang Y.N., Choi I.J., Kim D.K. (2015). Genetic characteristics of mitochondrial DNA was associated with colorectal carcinogenesis and its prognosis. PLoS ONE.

[38] López I., Oliveira L.P., Tucci P., Álvarez-Valín F., Coudry R.A., Marín M. (2012). Different mutation profiles associated to P53 accumulation in colorectal cancer. Gene.

[39] Tomonaga T., Izumi H., Yoshiura Y., Nishida C., Yatera K., Morimoto Y. (2021). Examination of Surfactant Protein D as a Biomarker for Evaluating Pulmonary Toxicity of Nanomaterials in Rat. Int. J. Mol. Sci..

[40] Obtulowicz T., Swoboda M., Speina E., Gackowski D., Rozalski R., Siomek A., Janik J., Janowska B., Ciesla J.M., Jawien A. (2010). Oxidative stress and 8-oxoguanine repair are enhanced in colon adenoma and carcinoma patients. Mutagenesis.

[41] Lei L., Zhang J., Decker E.A., Zhang G. (2021). Roles of Lipid Peroxidation-Derived Electrophiles in Pathogenesis of Colonic Inflammation and Colon Cancer. Front. Cell Dev. Biol..

[42] Wang W., Yang J., Edin M.L., Wang Y., Luo Y., Wan D., Yang H., Song C.Q., Xue W., Sanidad K.Z. (2019). Targeted Metabolomics Identifies the Cytochrome P450 Monooxygenase Eicosanoid Pathway as a Novel Therapeutic Target of Colon Tumorigenesis. Cancer Res..

[43] Wang Y., Wang W., Yang H., Shao D., Zhao X., Zhang G. (2019). Intraperitoneal injection of 4-hydroxynonenal (4-HNE), a lipid peroxidation product, exacerbates colonic inflammation through activation of Toll-like receptor 4 signaling. Free Radic. Biol. Med..

[44] Zhang W., Xiao S., Ahn D.U. (2013). Protein oxidation: Basic principles and implications for meat quality. Crit. Rev. Food Sci. Nutr..

[45] Yang H.Y., Chay K.O., Kwon J., Kwon S.O., Park Y.K., Lee T.H. (2013). Comparative proteomic analysis of cysteine oxidation in colorectal cancer patients. Mol. Cells.

[46] Cui Z., Cong M., Yin S., Li Y., Ye Y., Liu X., Tang J. (2024). Role of protein degradation systems in colorectal cancer. Cell Death Discov..

[47] Perillo B., Di Donato M., Pezone A., Di Zazzo E., Giovannelli P., Galasso G., Castoria G., Migliaccio A. (2020). ROS in cancer therapy: The bright side of the moon. Exp. Mol. Med..

[48] Gao L., Loveless J., Shay C., Teng Y. (2020). Targeting ROS-Mediated Crosstalk between Autophagy and Apoptosis in Cancer. Adv. Exp. Med. Biol..

[49] Huang Z., Gan S., Zhuang X., Chen Y., Lu L., Wang Y., Qi X., Feng Q., Huang Q., Du B. (2022). Artesunate Inhibits the Cell Growth in Colorectal Cancer by Promoting ROS-Dependent Cell Senescence and Autophagy. Cells.

[50] Thomas K.C., Parish S.L., Williams C.S. (2014). Healthcare expenditures for autism during times of school transition: Some vulnerable families fall behind. Matern. Child. Health J..

[51] Chang Y., Li F., Wang Z., Zhao Q., Wang Z., Han X., Xu Z., Yu C., Liu Y., Chang S. (2024). Oxidative balance score: A potential tool for reducing the risk of colorectal cancer and its subsites incidences. Front. Endocrinol..

[52] Baird L., Yamamoto M. (2020). The Molecular Mechanisms Regulating the KEAP1-NRF2 Pathway. Mol. Cell Biol..

[53] He L., He T., Farrar S., Ji L., Liu T., Ma X. (2017). Antioxidants Maintain Cellular Redox Homeostasis by Elimination of Reactive Oxygen Species. Cell Physiol. Biochem..

[54] Cheung K.L., Lee J.H., Khor T.O., Wu T.Y., Li G.X., Chan J., Yang C.S., Kong A.N. (2014). Nrf2 knockout enhances intestinal tumorigenesis in Apc(min/+) mice due to attenuation of anti-oxidative stress pathway while potentiates inflammation. Mol. Carcinog..

[55] Hammad A., Zheng Z.H., Gao Y., Namani A., Shi H.F., Tang X. (2019). Identification of novel Nrf2 target genes as prognostic biomarkers in colitis-associated colorectal cancer in Nrf2-deficient mice. Life Sci..

[56] Medoro A., Saso L., Scapagnini G., Davinelli S. (2023). NRF2 signaling pathway and telomere length in aging and age-related diseases. Mol. Cell. Biochem..

[57] Barnes R.P., Fouquerel E., Opresko P.L. (2019). The impact of oxidative DNA damage and stress on telomere homeostasis. Mech. Ageing Dev..

[58] Kansanen E., Kuosmanen S.M., Leinonen H., Levonen A.L. (2013). The Keap1-Nrf2 pathway: Mechanisms of activation and dysregulation in cancer. Redox Biol..

[59] Li J., Wang D., Liu Y., Zhou Y. (2022). Role of NRF2 in Colorectal Cancer Prevention and Treatment. Technol. Cancer Res. Treat..

[60] Wang G., Zhu Z.M., Wang K. (2024). Identification of ROS and KEAP1-related genes and verified targets of α-hederin induce cell death for CRC. Drug Dev. Res..

[61] Chang L.C., Fan C.W., Tseng W.K., Chen J.R., Hua C.C. (2022). The tumour/normal tissue ratio of Keap1 protein is a predictor for lymphovascular invasion in colorectal cancer: A correlation study between the Nrf2 and KRas pathways. Biomarkers.

[62] Horie Y., Suzuki T., Inoue J., Iso T., Wells G., Moore T.W., Mizushima T., Dinkova-Kostova A.T., Kasai T., Kamei T. (2021). Molecular basis for the disruption of Keap1-Nrf2 interaction via Hinge & Latch mechanism. Commun. Biol..

[63] Pouremamali F., Pouremamali A., Dadashpour M., Soozangar N., Jeddi F. (2022). An update of Nrf2 activators and inhibitors in cancer prevention/promotion. Cell Commun. Signal..

[64] Peng J., Song X., Yu W., Pan Y., Zhang Y., Jian H., He B. (2024). The role and mechanism of cinnamaldehyde in cancer. J. Food Drug Anal..

[65] Sato Y., Tsujinaka S., Miura T., Kitamura Y., Suzuki H., Shibata C. (2023). Inflammatory Bowel Disease and Colorectal Cancer: Epidemiology, Etiology, Surveillance, and Management. Cancers.

[66] Zhang Y.Z., Li Y.Y. (2014). Inflammatory bowel disease: Pathogenesis. World J. Gastroenterol..

[67] Veauthier B., Hornecker J.R. (2018). Crohn’s Disease: Diagnosis and Management. Am. Fam. Physician.

[68] Ordás I., Eckmann L., Talamini M., Baumgart D.C., Sandborn W.J. (2012). Ulcerative colitis. Lancet.

[69] Abu-Freha N., Cohen B., Gordon M., Weissmann S., Kestenbaum E.H., Vosko S., Abu-Tailakh M., Ben-Shoshan L., Cohen D.L., Shirin H. (2023). Colorectal cancer among inflammatory bowel disease patients: Risk factors and prevalence compared to the general population. Front. Med..

[70] Tian T., Wang Z., Zhang J. (2017). Pathomechanisms of Oxidative Stress in Inflammatory Bowel Disease and Potential Antioxidant Therapies. Oxid. Med. Cell. Longev..

[71] Kosaka T., Yoshino J., Inui K., Wakabayashi T., Kobayashi T., Watanabe S., Hayashi S., Hirokawa Y., Shiraishi T., Yamamoto T. (2009). Involvement of NAD(P)H:quinone oxidoreductase 1 and superoxide dismutase polymorphisms in ulcerative colitis. DNA Cell Biol..

[72] Zhou Y.J., Zhao B.L., Qian Z., Xu Y., Ding Y.Q. (2019). Association of Glutathione S-Transferase M1 null genotype with inflammatory bowel diseases: A systematic review and meta-analysis. Medicine.

[73] Hertervig E., Nilsson A., Seidegård J. (1994). The expression of glutathione transferase mu in patients with inflammatory bowel disease. Scand. J. Gastroenterol..

[74] Varzari A., Deyneko I.V., Tudor E., Turcan S. (2016). Polymorphisms of glutathione S-transferase and methylenetetrahydrofolate reductase genes in Moldavian patients with ulcerative colitis: Genotype-phenotype correlation. Meta Gene.

[75] Zhang Y., Yan T., Sun D., Xie C., Wang T., Liu X., Wang J., Wang Q., Luo Y., Wang P. (2020). Rutaecarpine inhibits KEAP1-NRF2 interaction to activate NRF2 and ameliorate dextran sulfate sodium-induced colitis. Free Radic. Biol. Med..

[76] Karban A., Hartman C., Eliakim R., Waterman M., Nesher S., Barnett-Griness O., Shamir R. (2007). Paraoxonase (PON)1 192R allele carriage is associated with reduced risk of inflammatory bowel disease. Dig. Dis. Sci..

[77] Keller D.S., Windsor A., Cohen R., Chand M. (2019). Colorectal cancer in inflammatory bowel disease: Review of the evidence. Tech. Coloproctol..

[78] Keshavarz Shahbaz S., Koushki K., Ayati S.H., Bland A.R., Bezsonov E.E., Sahebkar A. (2021). Inflammasomes and Colorectal Cancer. Cells.

[79] Patel M., Horgan P.G., McMillan D.C., Edwards J. (2018). NF-κB pathways in the development and progression of colorectal cancer. Transl. Res..

[80] Abais J.M., Xia M., Zhang Y., Boini K.M., Li P.L. (2015). Redox regulation of NLRP3 inflammasomes: ROS as trigger or effector?. Antioxid. Redox Signal..

[81] Zhong Z., Zhai Y., Liang S., Mori Y., Han R., Sutterwala F.S., Qiao L. (2013). TRPM2 links oxidative stress to NLRP3 inflammasome activation. Nat. Commun..

[82] Du Q., Wang Q., Fan H., Wang J., Liu X., Wang H., Wang Y., Hu R. (2016). Dietary cholesterol promotes AOM-induced colorectal cancer through activating the NLRP3 inflammasome. Biochem. Pharmacol..

[83] Yang Y., Wang H., Kouadir M., Song H., Shi F. (2019). Recent advances in the mechanisms of NLRP3 inflammasome activation and its inhibitors. Cell Death Dis..

[84] Chen Q.L., Yin H.R., He Q.Y., Wang Y. (2021). Targeting the NLRP3 inflammasome as new therapeutic avenue for inflammatory bowel disease. Biomed. Pharmacother..

[85] Deng Q., Geng Y., Zhao L., Li R., Zhang Z., Li K., Liang R., Shao X., Huang M., Zuo D. (2019). NLRP3 inflammasomes in macrophages drive colorectal cancer metastasis to the liver. Cancer Lett..

[86] Hassanzadeh P. (2011). Colorectal cancer and NF-κB signaling pathway. Gastroenterol. Hepatol. Bed Bench.

[87] Karin M. (1999). How NF-kappaB is activated: The role of the IkappaB kinase (IKK) complex. Oncogene.

[88] Sun S.C. (2017). The non-canonical NF-κB pathway in immunity and inflammation. Nat. Rev. Immunol..

[89] Yu H., Lin L., Zhang Z., Zhang H., Hu H. (2020). Targeting NF-κB pathway for the therapy of diseases: Mechanism and clinical study. Signal Transduct. Target. Ther..

[90] Mussbacher M., Salzmann M., Brostjan C., Hoesel B., Schoergenhofer C., Datler H., Hohensinner P., Basílio J., Petzelbauer P., Assinger A. (2019). Cell Type-Specific Roles of NF-κB Linking Inflammation and Thrombosis. Front. Immunol..

[91] Berkovich L., Gerber M., Katzav A., Kidron D., Avital S. (2022). NF-kappa B expression in resected specimen of colonic cancer is higher compared to its expression in inflammatory bowel diseases and polyps. Sci. Rep..

[92] Soleimani A., Rahmani F., Ferns G.A., Ryzhikov M., Avan A., Hassanian S.M. (2020). Role of the NF-κB signaling pathway in the pathogenesis of colorectal cancer. Gene.

[93] Park M.H., Hong J.T. (2016). Roles of NF-κB in Cancer and Inflammatory Diseases and Their Therapeutic Approaches. Cells.

[94] Rajitha B., Belalcazar A., Nagaraju G.P., Shaib W.L., Snyder J.P., Shoji M., Pattnaik S., Alam A., El-Rayes B.F. (2016). Inhibition of NF-κB translocation by curcumin analogs induces G0/G1 arrest and downregulates thymidylate synthase in colorectal cancer. Cancer Lett..

[95] Wang Y., Lu P., Zhang W., Du Q., Tang J., Wang H., Lu J., Hu R. (2016). GEN-27, a Newly Synthetic Isoflavonoid, Inhibits the Proliferation of Colon Cancer Cells in Inflammation Microenvironment by Suppressing NF-κB Pathway. Mediat. Inflamm..

[96] Martin M., Sun M., Motolani A., Lu T. (2021). The Pivotal Player: Components of NF-κB Pathway as Promising Biomarkers in Colorectal Cancer. Int. J. Mol. Sci..

[97] Gao W., Guo L., Yang Y., Wang Y., Xia S., Gong H., Zhang B.K., Yan M. (2021). Dissecting the Crosstalk Between Nrf2 and NF-κB Response Pathways in Drug-Induced Toxicity. Front. Cell Dev. Biol..

[98] Alcaraz M.J., Vicente A.M., Araico A., Dominguez J.N., Terencio M.C., Ferrándiz M.L. (2004). Role of nuclear factor-kappaB and heme oxygenase-1 in the mechanism of action of an anti-inflammatory chalcone derivative in RAW 264.7 cells. Br. J. Pharmacol..

[99] Huang Y., Ma T., Ye Z., Li H., Zhao Y., Chen W., Fu Y., Ye Z., Sun A., Li Z. (2018). Carbon monoxide (CO) inhibits hydrogen peroxide (H(2)O(2))-induced oxidative stress and the activation of NF-κB signaling in lens epithelial cells. Exp. Eye Res..

[100] Guinane C.M., Cotter P.D. (2013). Role of the gut microbiota in health and chronic gastrointestinal disease: Understanding a hidden metabolic organ. Therap. Adv. Gastroenterol..

[101] Bull M.J., Plummer N.T. (2014). Part 1: The Human Gut Microbiome in Health and Disease. Integr. Med..

[102] Afzaal M., Saeed F., Shah Y.A., Hussain M., Rabail R., Socol C.T., Hassoun A., Pateiro M., Lorenzo J.M., Rusu A.V. (2022). Human gut microbiota in health and disease: Unveiling the relationship. Front. Microbiol..

[103] Jandhyala S.M., Talukdar R., Subramanyam C., Vuyyuru H., Sasikala M., Nageshwar Reddy D. (2015). Role of the normal gut microbiota. World J. Gastroenterol..

[104] Hasan N., Yang H. (2019). Factors affecting the composition of the gut microbiota, and its modulation. PeerJ.

[105] Hamamah S., Amin A., Al-Kassir A.L., Chuang J., Covasa M. (2023). Dietary Fat Modulation of Gut Microbiota and Impact on Regulatory Pathways Controlling Food Intake. Nutrients.

[106] Flaig B., Garza R., Singh B., Hamamah S., Covasa M. (2023). Treatment of Dyslipidemia through Targeted Therapy of Gut Microbiota. Nutrients.

[107] Hamamah S., Hajnal A., Covasa M. (2022). Impact of Nutrition, Microbiota Transplant and Weight Loss Surgery on Dopaminergic Alterations in Parkinson’s Disease and Obesity. Int. J. Mol. Sci..

[108] Meng C., Bai C., Brown T.D., Hood L.E., Tian Q. (2018). Human Gut Microbiota and Gastrointestinal Cancer. Genom. Proteom. Bioinform..

[109] Peng X., Gao R., Ren J., Lu J., Ma X., Li P. (2023). Specific network information gain for detecting the critical state of colorectal cancer based on gut microbiome. Brief. Bioinform..

[110] Yachida S., Mizutani S., Shiroma H., Shiba S., Nakajima T., Sakamoto T., Watanabe H., Masuda K., Nishimoto Y., Kubo M. (2019). Metagenomic and metabolomic analyses reveal distinct stage-specific phenotypes of the gut microbiota in colorectal cancer. Nat. Med..

[111] Wang X., Jia Y., Wen L., Mu W., Wu X., Liu T., Liu X., Fang J., Luan Y., Chen P. (2021). Porphyromonas gingivalis Promotes Colorectal Carcinoma by Activating the Hematopoietic NLRP3 Inflammasome. Cancer Res..

[112] Xie Y.H., Gao Q.Y., Cai G.X., Sun X.M., Sun X.M., Zou T.H., Chen H.M., Yu S.Y., Qiu Y.W., Gu W.Q. (2017). Fecal Clostridium symbiosum for Noninvasive Detection of Early and Advanced Colorectal Cancer: Test and Validation Studies. EBioMedicine.

[113] Okumura S., Konishi Y., Narukawa M., Sugiura Y., Yoshimoto S., Arai Y., Sato S., Yoshida Y., Tsuji S., Uemura K. (2021). Gut bacteria identified in colorectal cancer patients promote tumourigenesis via butyrate secretion. Nat. Commun..

[114] Dikeocha I.J., Al-Kabsi A.M., Chiu H.T., Alshawsh M.A. (2022). Faecalibacterium prausnitzii Ameliorates Colorectal Tumorigenesis and Suppresses Proliferation of HCT116 Colorectal Cancer Cells. Biomedicines.

[115] Li Q., Hu W., Liu W.X., Zhao L.Y., Huang D., Liu X.D., Chan H., Zhang Y., Zeng J.D., Coker O.O. (2021). Streptococcus thermophilus Inhibits Colorectal Tumorigenesis Through Secreting β-Galactosidase. Gastroenterology.

[116] Qu R., Zhang Y., Ma Y., Zhou X., Sun L., Jiang C., Zhang Z., Fu W. (2023). Role of the Gut Microbiota and Its Metabolites in Tumorigenesis or Development of Colorectal Cancer. Adv. Sci..

[117] Prudhvi K., Jonnadula J., Kutti Sridharan G., Dominguez M. (2020). Systemic sclerosis with renal crisis and pericardial effusion. Clin. Case Rep..

[118] Wang N., Fang J.Y. (2023). Fusobacterium nucleatum, a key pathogenic factor and microbial biomarker for colorectal cancer. Trends Microbiol..

[119] Bing X., Wang Z., Wei F., Gao J., Xu D., Zhang L., Wang Y. (2020). Separation of m-Cresol from Coal Tar Model Oil Using Propylamine-Based Ionic Liquids: Extraction and Interaction Mechanism Exploration. ACS Omega.

[120] Bostanghadiri N., Razavi S., Shariati A., Talebi M., Mirkalantari S., Emami Razavi A., Darban-Sarokhalil D. (2023). Exploring the interplay between Fusobacterium nucleatum with the expression of microRNA, and inflammatory mediators in colorectal cancer. Front. Microbiol..

[121] Wang M., Wang Z., Lessing D.J., Guo M., Chu W. (2023). Fusobacterium nucleatum and its metabolite hydrogen sulfide alter gut microbiota composition and autophagy process and promote colorectal cancer progression. Microbiol. Spectr..

[122] Martin-Gallausiaux C., Salesse L., Garcia-Weber D., Marinelli L., Beguet-Crespel F., Brochard V., Le Gléau C., Jamet A., Doré J., Blottière H.M. (2024). Fusobacterium nucleatum promotes inflammatory and anti-apoptotic responses in colorectal cancer cells via ADP-heptose release and ALPK1/TIFA axis activation. Gut Microbes.

[123] Xu C., Fan L., Lin Y., Shen W., Qi Y., Zhang Y., Chen Z., Wang L., Long Y., Hou T. (2021). Fusobacterium nucleatum promotes colorectal cancer metastasis through miR-1322/CCL20 axis and M2 polarization. Gut Microbes.

[124] Chen S., Su T., Zhang Y., Lee A., He J., Ge Q., Wang L., Si J., Zhuo W., Wang L. (2020). Fusobacterium nucleatum promotes colorectal cancer metastasis by modulating KRT7-AS/KRT7. Gut Microbes.

[125] Chen S., Zhang L., Li M., Zhang Y., Sun M., Wang L., Lin J., Cui Y., Chen Q., Jin C. (2022). Fusobacterium nucleatum reduces METTL3-mediated m(6)A modification and contributes to colorectal cancer metastasis. Nat. Commun..

[126] Gao Y., Bi D., Xie R., Li M., Guo J., Liu H., Guo X., Fang J., Ding T., Zhu H. (2021). Fusobacterium nucleatum enhances the efficacy of PD-L1 blockade in colorectal cancer. Signal Transduct. Target. Ther..

[127] Li B., Wei Z., Wang Z., Xu F., Yang J., Lin B., Chen Y., Wenren H., Wu L., Guo X. (2024). Fusobacterium nucleatum induces oxaliplatin resistance by inhibiting ferroptosis through E-cadherin/β-catenin/GPX4 axis in colorectal cancer. Free Radic. Biol. Med..

[128] Wang N., Zhang L., Leng X.X., Xie Y.L., Kang Z.R., Zhao L.C., Song L.H., Zhou C.B., Fang J.Y. (2024). Fusobacterium nucleatum induces chemoresistance in colorectal cancer by inhibiting pyroptosis via the Hippo pathway. Gut Microbes.

[129] Zeng W., Pan J., Ye G. (2024). miR-135b Aggravates Fusobacterium nucleatum-Induced Cisplatin Resistance in Colorectal Cancer by Targeting KLF13. J. Microbiol..

[130] Karunakaran G., Yang Y., Tremblay V., Ning Z., Martin J., Belaouad A., Figeys D., Brunzelle J.S., Giguere P.M., Stintzi A. (2022). Structural analysis of Atopobium parvulum SufS cysteine desulfurase linked to Crohn’s disease. FEBS Lett..

[131] Coker O.O., Liu C., Wu W.K.K., Wong S.H., Jia W., Sung J.J.Y., Yu J. (2022). Altered gut metabolites and microbiota interactions are implicated in colorectal carcinogenesis and can be non-invasive diagnostic biomarkers. Microbiome.

[132] Hasan R., Bose S., Roy R., Paul D., Rawat S., Nilwe P., Chauhan N.K., Choudhury S. (2022). Tumor tissue-specific bacterial biomarker panel for colorectal cancer: Bacteroides massiliensis, Alistipes species, Alistipes onderdonkii, Bifidobacterium pseudocatenulatum, Corynebacterium appendicis. Arch. Microbiol..

[133] Kerdreux M., Edin S., Löwenmark T., Bronnec V., Löfgren-Burström A., Zingmark C., Ljuslinder I., Palmqvist R., Ling A. (2023). Porphyromonas gingivalis in Colorectal Cancer and its Association to Patient Prognosis. J. Cancer.

[134] Sorolla M.A., Hidalgo I., Sorolla A., Montal R., Pallisé O., Salud A., Parisi E. (2021). Microenvironmental Reactive Oxygen Species in Colorectal Cancer: Involved Processes and Therapeutic Opportunities. Cancers.

[135] Irrazabal T., Thakur B.K., Kang M., Malaise Y., Streutker C., Wong E.O.Y., Copeland J., Gryfe R., Guttman D.S., Navarre W.W. (2020). Limiting oxidative DNA damage reduces microbe-induced colitis-associated colorectal cancer. Nat. Commun..

[136] Dumitrescu L., Popescu-Olaru I., Cozma L., Tulbă D., Hinescu M.E., Ceafalan L.C., Gherghiceanu M., Popescu B.O. (2018). Oxidative Stress and the Microbiota-Gut-Brain Axis. Oxid. Med. Cell. Longev..

[137] Aceto G.M., Catalano T., Curia M.C. (2020). Molecular Aspects of Colorectal Adenomas: The Interplay among Microenvironment, Oxidative Stress, and Predisposition. Biomed. Res. Int..

[138] Schmitt M., Greten F.R. (2021). The inflammatory pathogenesis of colorectal cancer. Nat. Rev. Immunol..

[139] Allam-Ndoul B., Castonguay-Paradis S., Veilleux A. (2020). Gut Microbiota and Intestinal Trans-Epithelial Permeability. Int. J. Mol. Sci..

[140] Goodwin A.C., Destefano Shields C.E., Wu S., Huso D.L., Wu X., Murray-Stewart T.R., Hacker-Prietz A., Rabizadeh S., Woster P.M., Sears C.L. (2011). Polyamine catabolism contributes to enterotoxigenic Bacteroides fragilis-induced colon tumorigenesis. Proc. Natl. Acad. Sci. USA.

[141] Tsoi H., Chu E.S.H., Zhang X., Sheng J., Nakatsu G., Ng S.C., Chan A.W.H., Chan F.K.L., Sung J.J.Y., Yu J. (2017). Peptostreptococcus anaerobius Induces Intracellular Cholesterol Biosynthesis in Colon Cells to Induce Proliferation and Causes Dysplasia in Mice. Gastroenterology.

[142] Huycke M.M., Moore D.R. (2002). In vivo production of hydroxyl radical by Enterococcus faecalis colonizing the intestinal tract using aromatic hydroxylation. Free Radic. Biol. Med..

[143] Sears C.L., Geis A.L., Housseau F. (2014). Bacteroides fragilis subverts mucosal biology: From symbiont to colon carcinogenesis. J. Clin. Investig..

[144] Rajkumar S.D., Gunabooshanam B., Joseph L.D., D’Cruze L. (2022). Utility of immunohistochemical expression of E-cadherin in colorectal carcinoma. Prz. Gastroenterol..

[145] Cervelli M., Amendola R., Polticelli F., Mariottini P. (2012). Spermine oxidase: Ten years after. Amino Acids.

[146] Chaturvedi R., Asim M., Romero-Gallo J., Barry D.P., Hoge S., de Sablet T., Delgado A.G., Wroblewski L.E., Piazuelo M.B., Yan F. (2011). Spermine oxidase mediates the gastric cancer risk associated with Helicobacter pylori CagA. Gastroenterology.

[147] Uemura T., Tanaka Y., Higashi K., Miyamori D., Takasaka T., Nagano T., Toida T., Yoshimoto K., Igarashi K., Ikegaya H. (2013). Acetaldehyde-induced cytotoxicity involves induction of spermine oxidase at the transcriptional level. Toxicology.

[148] Cervelli M., Bellavia G., Fratini E., Amendola R., Polticelli F., Barba M., Federico R., Signore F., Gucciardo G., Grillo R. (2010). Spermine oxidase (SMO) activity in breast tumor tissues and biochemical analysis of the anticancer spermine analogues BENSpm and CPENSpm. BMC Cancer.

[149] Sun L., Yang J., Qin Y., Wang Y., Wu H., Zhou Y., Cao C. (2019). Discovery and antitumor evaluation of novel inhibitors of spermine oxidase. J. Enzyme Inhib. Med. Chem..

[150] Wang X., Allen T.D., May R.J., Lightfoot S., Houchen C.W., Huycke M.M. (2008). Enterococcus faecalis induces aneuploidy and tetraploidy in colonic epithelial cells through a bystander effect. Cancer Res..

[151] Scheible M., Nguyen C.T., Luong T.T., Lee J.H., Chen Y.W., Chang C., Wittchen M., Camacho M.I., Tiner B.L., Wu C. (2022). The Fused Methionine Sulfoxide Reductase MsrAB Promotes Oxidative Stress Defense and Bacterial Virulence in Fusobacterium nucleatum. mBio.

[152] Silva V.L., Diniz C.G., Cara D.C., Santos S.G., Nicoli J.R., Carvalho M.A., Farias L.M. (2005). Enhanced pathogenicity of Fusobacterium nucleatum adapted to oxidative stress. Microb. Pathog..

[153] Frances Nakamya M., Ayoola M.B., Shack L.A., Mohamed M., Swiatlo E., Nanduri B. (2021). Arginine Decarboxylase Is Essential for Pneumococcal Stress Responses. Pathogens.

[154] McDowell R., Perrott S., Murchie P., Cardwell C., Hughes C., Samuel L. (2022). Oral antibiotic use and early-onset colorectal cancer: Findings from a case-control study using a national clinical database. Br. J. Cancer.

[155] Aneke-Nash C., Yoon G., Du M., Liang P. (2021). Antibiotic use and colorectal neoplasia: A systematic review and meta-analysis. BMJ Open Gastroenterol..

[156] Voskarides K. (2022). An evolutionary explanation for antibiotics’ association with increased colon cancer risk. Evol. Med. Public. Health.

[157] Kim M., Yun S.Y., Lee Y., Lee H., Yong D., Lee K. (2022). Clinical Differences in Patients Infected with Fusobacterium and Antimicrobial Susceptibility of Fusobacterium Isolates Recovered at a Tertiary-Care Hospital in Korea. Ann. Lab. Med..

[158] Ju Y., Liu K., Ma G., Zhu B., Wang H., Hu Z., Zhao J., Zhang L., Cui K., He X.R. (2023). Bacterial antibiotic resistance among cancer inpatients in China: 2016-20. Qjm.

[159] Oberc A.M., Fiebig-Comyn A.A., Tsai C.N., Elhenawy W., Coombes B.K. (2019). Antibiotics Potentiate Adherent-Invasive *E. coli* Infection and Expansion. Inflamm. Bowel Dis..

[160] Hamoya T., Miyamoto S., Tomono S., Fujii G., Nakanishi R., Komiya M., Tamura S., Fujimoto K., Toshima J., Wakabayashi K. (2017). Chemopreventive effects of a low-side-effect antibiotic drug, erythromycin, on mouse intestinal tumors. J. Clin. Biochem. Nutr..

[161] Wang D., Dubois R.N. (2010). The role of COX-2 in intestinal inflammation and colorectal cancer. Oncogene.

[162] Li S., Liu J., Zheng X., Ren L., Yang Y., Li W., Fu W., Wang J., Du G. (2021). Tumorigenic bacteria in colorectal cancer: Mechanisms and treatments. Cancer Biol. Med..

[163] Heidari A., Emami M.H., Maghool F., Mohammadzadeh S., Kadkhodaei Elyaderani P., Safari T., Fahim A., Kamali Dolatabadi R. (2024). Molecular epidemiology, antibiotic resistance profile and frequency of integron 1 and 2 in adherent-invasive *Escherichia coli* isolates of colorectal cancer patients. Front. Microbiol..

[164] Bullman S., Pedamallu C.S., Sicinska E., Clancy T.E., Zhang X., Cai D., Neuberg D., Huang K., Guevara F., Nelson T. (2017). Analysis of Fusobacterium persistence and antibiotic response in colorectal cancer. Science.

[165] DeStefano Shields C.E., Van Meerbeke S.W., Housseau F., Wang H., Huso D.L., Casero R.A., O’Hagan H.M., Sears C.L. (2016). Reduction of Murine Colon Tumorigenesis Driven by Enterotoxigenic Bacteroides fragilis Using Cefoxitin Treatment. J. Infect. Dis..

[166] Yang Q., Wang B., Zheng Q., Li H., Meng X., Zhou F., Zhang L. (2023). A Review of Gut Microbiota-Derived Metabolites in Tumor Progression and Cancer Therapy. Adv. Sci..

[167] Avuthu N., Guda C. (2022). Meta-Analysis of Altered Gut Microbiota Reveals Microbial and Metabolic Biomarkers for Colorectal Cancer. Microbiol. Spectr..

[168] Wu Y., Jiao N., Zhu R., Zhang Y., Wu D., Wang A.J., Fang S., Tao L., Li Y., Cheng S. (2021). Identification of microbial markers across populations in early detection of colorectal cancer. Nat. Commun..

[169] Nguyen T.T., Ung T.T., Kim N.H., Jung Y.D. (2018). Role of bile acids in colon carcinogenesis. World J. Clin. Cases.

[170] Duizer C., de Zoete M.R. (2023). The Role of Microbiota-Derived Metabolites in Colorectal Cancer. Int. J. Mol. Sci..

[171] Xiong R.G., Zhou D.D., Wu S.X., Huang S.Y., Saimaiti A., Yang Z.J., Shang A., Zhao C.N., Gan R.Y., Li H.B. (2022). Health Benefits and Side Effects of Short-Chain Fatty Acids. Foods.

[172] Louis P., Flint H.J. (2017). Formation of propionate and butyrate by the human colonic microbiota. Environ. Microbiol..

[173] Zhang D., Jian Y.P., Zhang Y.N., Li Y., Gu L.T., Sun H.H., Liu M.D., Zhou H.L., Wang Y.S., Xu Z.X. (2023). Short-chain fatty acids in diseases. Cell Commun. Signal..

[174] Alvandi E., Wong W.K.M., Joglekar M.V., Spring K.J., Hardikar A.A. (2022). Short-chain fatty acid concentrations in the incidence and risk-stratification of colorectal cancer: A systematic review and meta-analysis. BMC Med..

[175] Wu Q.L., Fang X.T., Wan X.X., Ding Q.Y., Zhang Y.J., Ji L., Lou Y.L., Li X. (2024). Fusobacterium nucleatum-induced imbalance in microbiome-derived butyric acid levels promotes the occurrence and development of colorectal cancer. World J. Gastroenterol..

[176] Son M.Y., Cho H.S. (2023). Anticancer Effects of Gut Microbiota-Derived Short-Chain Fatty Acids in Cancers. J. Microbiol. Biotechnol..

[177] Liu P., Wang Y., Yang G., Zhang Q., Meng L., Xin Y., Jiang X. (2021). The role of short-chain fatty acids in intestinal barrier function, inflammation, oxidative stress, and colonic carcinogenesis. Pharmacol. Res..

[178] Hamer H.M., Jonkers D.M., Bast A., Vanhoutvin S.A., Fischer M.A., Kodde A., Troost F.J., Venema K., Brummer R.J. (2009). Butyrate modulates oxidative stress in the colonic mucosa of healthy humans. Clin. Nutr..

[179] Huang W., Guo H.L., Deng X., Zhu T.T., Xiong J.F., Xu Y.H., Xu Y. (2017). Short-Chain Fatty Acids Inhibit Oxidative Stress and Inflammation in Mesangial Cells Induced by High Glucose and Lipopolysaccharide. Exp. Clin. Endocrinol. Diabetes.

[180] Zhang W., Wang W., Xu M., Xie H., Pu Z. (2021). GPR43 regulation of mitochondrial damage to alleviate inflammatory reaction in sepsis. Aging (Albany NY).

[181] Masui R., Sasaki M., Funaki Y., Ogasawara N., Mizuno M., Iida A., Izawa S., Kondo Y., Ito Y., Tamura Y. (2013). G protein-coupled receptor 43 moderates gut inflammation through cytokine regulation from mononuclear cells. Inflamm. Bowel Dis..

[182] Hu Y., Le Leu R.K., Christophersen C.T., Somashekar R., Conlon M.A., Meng X.Q., Winter J.M., Woodman R.J., McKinnon R., Young G.P. (2016). Manipulation of the gut microbiota using resistant starch is associated with protection against colitis-associated colorectal cancer in rats. Carcinogenesis.

[183] Kim H.J., Bae S.C. (2011). Histone deacetylase inhibitors: Molecular mechanisms of action and clinical trials as anti-cancer drugs. Am. J. Transl. Res..

[184] Fawad J.A., Luzader D.H., Hanson G.F., Moutinho T.J., McKinney C.A., Mitchell P.G., Brown-Steinke K., Kumar A., Park M., Lee S. (2022). Histone Deacetylase Inhibition by Gut Microbe-Generated Short-Chain Fatty Acids Entrains Intestinal Epithelial Circadian Rhythms. Gastroenterology.

[185] Place R.F., Noonan E.J., Giardina C. (2005). HDAC inhibition prevents NF-kappa B activation by suppressing proteasome activity: Down-regulation of proteasome subunit expression stabilizes I kappa B alpha. Biochem. Pharmacol..

[186] Feng Y., Wang Y., Wang P., Huang Y., Wang F. (2018). Short-Chain Fatty Acids Manifest Stimulative and Protective Effects on Intestinal Barrier Function Through the Inhibition of NLRP3 Inflammasome and Autophagy. Cell Physiol. Biochem..

[187] Scharlau D., Borowicki A., Habermann N., Hofmann T., Klenow S., Miene C., Munjal U., Stein K., Glei M. (2009). Mechanisms of primary cancer prevention by butyrate and other products formed during gut flora-mediated fermentation of dietary fibre. Mutat. Res..

[188] Salvi P.S., Cowles R.A. (2021). Butyrate and the Intestinal Epithelium: Modulation of Proliferation and Inflammation in Homeostasis and Disease. Cells.

[189] Siavoshian S., Blottiere H.M., Cherbut C., Galmiche J.P. (1997). Butyrate stimulates cyclin D and p21 and inhibits cyclin-dependent kinase 2 expression in HT-29 colonic epithelial cells. Biochem. Biophys. Res. Commun..

[190] Wang L., Shannar A.A.F., Wu R., Chou P., Sarwar M.S., Kuo H.C., Peter R.M., Wang Y., Su X., Kong A.N. (2022). Butyrate Drives Metabolic Rewiring and Epigenetic Reprogramming in Human Colon Cancer Cells. Mol. Nutr. Food Res..

[191] Guo W., Liu J., Sun J., Gong Q., Ma H., Kan X., Cao Y., Wang J., Fu S. (2020). Butyrate alleviates oxidative stress by regulating NRF2 nuclear accumulation and H3K9/14 acetylation via GPR109A in bovine mammary epithelial cells and mammary glands. Free Radic. Biol. Med..

[192] Lee Y.J., Kim W.I., Bae J.H., Cho M.K., Lee S.H., Nam H.S., Choi I.H., Cho S.W. (2020). Overexpression of Nrf2 promotes colon cancer progression via ERK and AKT signaling pathways. Ann. Surg. Treat. Res..

[193] Lau A., Villeneuve N.F., Sun Z., Wong P.K., Zhang D.D. (2008). Dual roles of Nrf2 in cancer. Pharmacol. Res..

[194] Gonzalez-Donquiles C., Alonso-Molero J., Fernandez-Villa T., Vilorio-Marqués L., Molina A.J., Martín V. (2017). The NRF2 transcription factor plays a dual role in colorectal cancer: A systematic review. PLoS ONE.

[195] Hodgkinson K., El Abbar F., Dobranowski P., Manoogian J., Butcher J., Figeys D., Mack D., Stintzi A. (2023). Butyrate’s role in human health and the current progress towards its clinical application to treat gastrointestinal disease. Clin. Nutr..

[196] Donohoe D.R., Collins L.B., Wali A., Bigler R., Sun W., Bultman S.J. (2012). The Warburg effect dictates the mechanism of butyrate-mediated histone acetylation and cell proliferation. Mol. Cell.

[197] Eslami M., Sadrifar S., Karbalaei M., Keikha M., Kobyliak N.M., Yousefi B. (2020). Importance of the Microbiota Inhibitory Mechanism on the Warburg Effect in Colorectal Cancer Cells. J. Gastrointest. Cancer.

[198] Huang C., Deng W., Xu H.Z., Zhou C., Zhang F., Chen J., Bao Q., Zhou X., Liu M., Li J. (2023). Short-chain fatty acids reprogram metabolic profiles with the induction of reactive oxygen species production in human colorectal adenocarcinoma cells. Comput. Struct. Biotechnol. J..

[199] Tailor D., Hahm E.R., Kale R.K., Singh S.V., Singh R.P. (2014). Sodium butyrate induces DRP1-mediated mitochondrial fusion and apoptosis in human colorectal cancer cells. Mitochondrion.

[200] Galadari S., Rahman A., Pallichankandy S., Thayyullathil F. (2017). Reactive oxygen species and cancer paradox: To promote or to suppress?. Free Radic. Biol. Med..

[201] Chiang J.Y. (2013). Bile acid metabolism and signaling. Compr. Physiol..

[202] di Gregorio M.C., Cautela J., Galantini L. (2021). Physiology and Physical Chemistry of Bile Acids. Int. J. Mol. Sci..

[203] Durník R., Šindlerová L., Babica P., Jurček O. (2022). Bile Acids Transporters of Enterohepatic Circulation for Targeted Drug Delivery. Molecules.

[204] Staley C., Weingarden A.R., Khoruts A., Sadowsky M.J. (2017). Interaction of gut microbiota with bile acid metabolism and its influence on disease states. Appl. Microbiol. Biotechnol..

[205] Yntema T., Koonen D.P.Y., Kuipers F. (2023). Emerging Roles of Gut Microbial Modulation of Bile Acid Composition in the Etiology of Cardiovascular Diseases. Nutrients.

[206] Harris S.C., Devendran S., Méndez-García C., Mythen S.M., Wright C.L., Fields C.J., Hernandez A.G., Cann I., Hylemon P.B., Ridlon J.M. (2018). Bile acid oxidation by Eggerthella lenta strains C592 and DSM 2243(T). Gut Microbes.

[207] Doden H., Sallam L.A., Devendran S., Ly L., Doden G., Daniel S.L., Alves J.M.P., Ridlon J.M. (2018). Metabolism of Oxo-Bile Acids and Characterization of Recombinant 12α-Hydroxysteroid Dehydrogenases from Bile Acid 7α-Dehydroxylating Human Gut Bacteria. Appl. Environ. Microbiol..

[208] Dvorak K., Payne C.M., Chavarria M., Ramsey L., Dvorakova B., Bernstein H., Holubec H., Sampliner R.E., Guy N., Condon A. (2007). Bile acids in combination with low pH induce oxidative stress and oxidative DNA damage: Relevance to the pathogenesis of Barrett’s oesophagus. Gut.

[209] Payne C.M., Weber C., Crowley-Skillicorn C., Dvorak K., Bernstein H., Bernstein C., Holubec H., Dvorakova B., Garewal H. (2007). Deoxycholate induces mitochondrial oxidative stress and activates NF-kappaB through multiple mechanisms in HCT-116 colon epithelial cells. Carcinogenesis.

[210] Ignacio Barrasa J., Olmo N., Pérez-Ramos P., Santiago-Gómez A., Lecona E., Turnay J., Antonia Lizarbe M. (2011). Deoxycholic and chenodeoxycholic bile acids induce apoptosis via oxidative stress in human colon adenocarcinoma cells. Apoptosis.

[211] Ajouz H., Mukherji D., Shamseddine A. (2014). Secondary bile acids: An underrecognized cause of colon cancer. World J. Surg. Oncol..

[212] Degirolamo C., Modica S., Palasciano G., Moschetta A. (2011). Bile acids and colon cancer: Solving the puzzle with nuclear receptors. Trends Mol. Med..

[213] Glinghammar B., Inoue H., Rafter J.J. (2002). Deoxycholic acid causes DNA damage in colonic cells with subsequent induction of caspases, COX-2 promoter activity and the transcription factors NF-kB and AP-1. Carcinogenesis.

[214] Kundu S., Kumar S., Bajaj A. (2015). Cross-talk between bile acids and gastrointestinal tract for progression and development of cancer and its therapeutic implications. IUBMB Life.

[215] Wang W., Mani A.M., Wu Z.H. (2017). DNA damage-induced nuclear factor-kappa B activation and its roles in cancer progression. J. Cancer Metastasis Treat..

[216] Orozco-Aguilar J., Simon F., Cabello-Verrugio C. (2021). Redox-Dependent Effects in the Physiopathological Role of Bile Acids. Oxid. Med. Cell. Longev..

[217] Lee H.Y., Crawley S., Hokari R., Kwon S., Kim Y.S. (2010). Bile acid regulates MUC2 transcription in colon cancer cells via positive EGFR/PKC/Ras/ERK/CREB, PI3K/Akt/IkappaB/NF-kappaB and p38/MSK1/CREB pathways and negative JNK/c-Jun/AP-1 pathway. Int. J. Oncol..

[218] Crowley-Weber C.L., Payne C.M., Gleason-Guzman M., Watts G.S., Futscher B., Waltmire C.N., Crowley C., Dvorakova K., Bernstein C., Craven M. (2002). Development and molecular characterization of HCT-116 cell lines resistant to the tumor promoter and multiple stress-inducer, deoxycholate. Carcinogenesis.

[219] Huang W.K., Hsu H.C., Liu J.R., Yang T.S., Chen J.S., Chang J.W., Lin Y.C., Yu K.H., Kuo C.F., See L.C. (2016). The Association of Ursodeoxycholic Acid Use With Colorectal Cancer Risk: A Nationwide Cohort Study. Medicine.

[220] Zhang H., Xu H., Zhang C., Tang Q., Bi F. (2021). Ursodeoxycholic acid suppresses the malignant progression of colorectal cancer through TGR5-YAP axis. Cell Death Discov..

[221] Kim E.K., Cho J.H., Kim E., Kim Y.J. (2017). Ursodeoxycholic acid inhibits the proliferation of colon cancer cells by regulating oxidative stress and cancer stem-like cell growth. PLoS ONE.

[222] Pearson T., Caporaso J.G., Yellowhair M., Bokulich N.A., Padi M., Roe D.J., Wertheim B.C., Linhart M., Martinez J.A., Bilagody C. (2019). Effects of ursodeoxycholic acid on the gut microbiome and colorectal adenoma development. Cancer Med..

[223] He Q., Wu J., Ke J., Zhang Q., Zeng W., Luo Z., Gong J., Chen Y., He Z., Lan P. (2023). Therapeutic role of ursodeoxycholic acid in colitis-associated cancer via gut microbiota modulation. Mol. Ther..

[224] Zhang Y., Jiang R., Zheng X., Lei S., Huang F., Xie G., Kwee S., Yu H., Farrar C., Sun B. (2019). Ursodeoxycholic acid accelerates bile acid enterohepatic circulation. Br. J. Pharmacol..

[225] Amaral J.D., Viana R.J., Ramalho R.M., Steer C.J., Rodrigues C.M. (2009). Bile acids: Regulation of apoptosis by ursodeoxycholic acid. J. Lipid Res..

[226] Guo X., Okpara E.S., Hu W., Yan C., Wang Y., Liang Q., Chiang J.Y.L., Han S. (2022). Interactive Relationships between Intestinal Flora and Bile Acids. Int. J. Mol. Sci..

[227] Yu Z.L., Zhang L.Y., Jiang X.M., Xue C.H., Chi N., Zhang T.T., Wang Y.M. (2020). Effects of dietary choline, betaine, and L-carnitine on the generation of trimethylamine-N-oxide in healthy mice. J. Food Sci..

[228] Rath S., Rud T., Pieper D.H., Vital M. (2019). Potential TMA-Producing Bacteria Are Ubiquitously Found in Mammalia. Front. Microbiol..

[229] Zhen J., Zhou Z., He M., Han H.X., Lv E.H., Wen P.B., Liu X., Wang Y.T., Cai X.C., Tian J.Q. (2023). The gut microbial metabolite trimethylamine N-oxide and cardiovascular diseases. Front. Endocrinol..

[230] Shih D.M., Wang Z., Lee R., Meng Y., Che N., Charugundla S., Qi H., Wu J., Pan C., Brown J.M. (2015). Flavin containing monooxygenase 3 exerts broad effects on glucose and lipid metabolism and atherosclerosis. J. Lipid Res..

[231] Jalandra R., Dalal N., Yadav A.K., Verma D., Sharma M., Singh R., Khosla A., Kumar A., Solanki P.R. (2021). Emerging role of trimethylamine-N-oxide (TMAO) in colorectal cancer. Appl. Microbiol. Biotechnol..

[232] Sun X., Jiao X., Ma Y., Liu Y., Zhang L., He Y., Chen Y. (2016). Trimethylamine N-oxide induces inflammation and endothelial dysfunction in human umbilical vein endothelial cells via activating ROS-TXNIP-NLRP3 inflammasome. Biochem. Biophys. Res. Commun..

[233] Boini K.M., Hussain T., Li P.L., Koka S. (2017). Trimethylamine-N-Oxide Instigates NLRP3 Inflammasome Activation and Endothelial Dysfunction. Cell Physiol. Biochem..

[234] Farhangi M.A., Vajdi M. (2020). Novel findings of the association between gut microbiota-derived metabolite trimethylamine N-oxide and inflammation: Results from a systematic review and dose-response meta-analysis. Crit. Rev. Food Sci. Nutr..

[235] Yue C., Yang X., Li J., Chen X., Zhao X., Chen Y., Wen Y. (2017). Trimethylamine N-oxide prime NLRP3 inflammasome via inhibiting ATG16L1-induced autophagy in colonic epithelial cells. Biochem. Biophys. Res. Commun..

[236] Wu Y., Rong X., Pan M., Wang T., Yang H., Chen X., Xiao Z., Zhao C. (2022). Integrated Analysis Reveals the Gut Microbial Metabolite TMAO Promotes Inflammatory Hepatocellular Carcinoma by Upregulating POSTN. Front. Cell Dev. Biol..

[237] Mirji G., Worth A., Bhat S.A., El Sayed M., Kannan T., Goldman A.R., Tang H.Y., Liu Q., Auslander N., Dang C.V. (2022). The microbiome-derived metabolite TMAO drives immune activation and boosts responses to immune checkpoint blockade in pancreatic cancer. Sci. Immunol..

[238] Yang S., Li X., Yang F., Zhao R., Pan X., Liang J., Tian L., Li X., Liu L., Xing Y. (2019). Gut Microbiota-Dependent Marker TMAO in Promoting Cardiovascular Disease: Inflammation Mechanism, Clinical Prognostic, and Potential as a Therapeutic Target. Front. Pharmacol..

[239] Zhao J., Zhao F., Yuan J., Liu H., Wang Y. (2023). Gut microbiota metabolites, redox status, and the related regulatory effects of probiotics. Heliyon.

[240] Ding S., Hu C., Fang J., Liu G. (2020). The Protective Role of Probiotics against Colorectal Cancer. Oxid. Med. Cell. Longev..

[241] Ballini A., Santacroce L., Cantore S., Bottalico L., Dipalma G., Topi S., Saini R., De Vito D., Inchingolo F. (2019). Probiotics Efficacy on Oxidative Stress Values in Inflammatory Bowel Disease: A Randomized Double-Blinded Placebo-Controlled Pilot Study. Endocr. Metab. Immune Disord. Drug Targets.

[242] Mendes M.C.S., Paulino D.S., Brambilla S.R., Camargo J.A., Persinoti G.F., Carvalheira J.B.C. (2018). Microbiota modification by probiotic supplementation reduces colitis associated colon cancer in mice. World J. Gastroenterol..

[243] Feng T., Wang J. (2020). Oxidative stress tolerance and antioxidant capacity of lactic acid bacteria as probiotic: A systematic review. Gut Microbes.

[244] Bell H.N., Rebernick R.J., Goyert J., Singhal R., Kuljanin M., Kerk S.A., Huang W., Das N.K., Andren A., Solanki S. (2022). Reuterin in the healthy gut microbiome suppresses colorectal cancer growth through altering redox balance. Cancer Cell.

[245] Aghamohammad S., Sepehr A., Miri S.T., Najafi S., Rohani M., Pourshafiea M.R. (2022). The effects of the probiotic cocktail on modulation of the NF-kB and JAK/STAT signaling pathways involved in the inflammatory response in bowel disease model. BMC Immunol..

[246] Kong Y., Olejar K.J., On S.L.W., Chelikani V. (2020). The Potential of Lactobacillus spp. for Modulating Oxidative Stress in the Gastrointestinal Tract. Antioxidants.

[247] Rocha-Ramírez L.M., Pérez-Solano R.A., Castañón-Alonso S.L., Moreno Guerrero S.S., Ramírez Pacheco A., García Garibay M., Eslava C. (2017). Probiotic Lactobacillus Strains Stimulate the Inflammatory Response and Activate Human Macrophages. J. Immunol. Res..

[248] Finamore A., Ambra R., Nobili F., Garaguso I., Raguzzini A., Serafini M. (2018). Redox Role of Lactobacillus casei Shirota Against the Cellular Damage Induced by 2,2′-Azobis (2-Amidinopropane) Dihydrochloride-Induced Oxidative and Inflammatory Stress in Enterocytes-Like Epithelial Cells. Front. Immunol..

[249] Riaz Rajoka M.S., Thirumdas R., Mehwish H.M., Umair M., Khurshid M., Hayat H.F., Phimolsiripol Y., Pallarés N., Martí-Quijal F.J., Barba F.J. (2021). Role of Food Antioxidants in Modulating Gut Microbial Communities: Novel Understandings in Intestinal Oxidative Stress Damage and Their Impact on Host Health. Antioxidants.

[250] Al-Numair K.S., Waly M.I., Ali A., Essa M.M., Farhat M.F., Alsaif M.A. (2011). Dietary folate protects against azoxymethane-induced aberrant crypt foci development and oxidative stress in rat colon. Exp. Biol. Med..

[251] Kang X., Liu C., Ding Y., Ni Y., Ji F., Lau H.C.H., Jiang L., Sung J.J., Wong S.H., Yu J. (2023). Roseburia intestinalis generated butyrate boosts anti-PD-1 efficacy in colorectal cancer by activating cytotoxic CD8(+) T cells. Gut.

[252] Talwalkar A., Kailasapathy K. (2004). The role of oxygen in the viability of probiotic bacteria with reference to *L. acidophilus* and *Bifidobacterium* spp.. Curr. Issues Intest. Microbiol..

[253] Goffin P., Muscariello L., Lorquet F., Stukkens A., Prozzi D., Sacco M., Kleerebezem M., Hols P. (2006). Involvement of pyruvate oxidase activity and acetate production in the survival of Lactobacillus plantarum during the stationary phase of aerobic growth. Appl. Environ. Microbiol..

[254] Shehata A.I., Soliman A.A., Ahmed H.A., Gewaily M.S., Amer A.A., Shukry M., Abdel-Latif H.M.R. (2024). Evaluation of different probiotics on growth, body composition, antioxidant capacity, and histoarchitecture of Mugil capito. Sci. Rep..

[255] de Moreno de LeBlanc A., LeBlanc J.G., Perdigón G., Miyoshi A., Langella P., Azevedo V., Sesma F. (2008). Oral administration of a catalase-producing Lactococcus lactis can prevent a chemically induced colon cancer in mice. J. Med. Microbiol..

[256] Deepak V., Sundar W.A., Pandian S.R.K., Sivasubramaniam S.D., Hariharan N., Sundar K. (2021). Exopolysaccharides from Lactobacillus acidophilus modulates the antioxidant status of 1,2-dimethyl hydrazine-induced colon cancer rat model. 3 Biotech.

[257] Venkatachalam K., Vinayagam R., Arokia Vijaya Anand M., Isa N.M., Ponnaiyan R. (2020). Biochemical and molecular aspects of 1,2-dimethylhydrazine (DMH)-induced colon carcinogenesis: A review. Toxicol. Res..

[258] Sengül N., Işık S., Aslım B., Uçar G., Demirbağ A.E. (2011). The effect of exopolysaccharide-producing probiotic strains on gut oxidative damage in experimental colitis. Dig. Dis. Sci..

[259] Kwun S.Y., Yoon J.A., Kim G.Y., Bae Y.W., Park E.H., Kim M.D. (2024). Isolation of a Potential Probiotic Levilactobacillus brevis and Evaluation of Its Exopolysaccharide for Antioxidant and α-Glucosidase Inhibitory Activities. J. Microbiol. Biotechnol..

[260] Sadeghi M., Haghshenas B., Nami Y. (2024). Bifidobacterium exopolysaccharides: New insights into engineering strategies, physicochemical functions, and immunomodulatory effects on host health. Front. Microbiol..

[261] de Oliveira C.S., Baptistella M.M., Siqueira A.P., Carvalho M.O., Ramos L.F., Souto B.S., de Almeida L.A., Dos Santos E.G., Novaes R.D., Nogueira E.S.C. (2023). Combination of vitamin D and probiotics inhibits chemically induced colorectal carcinogenesis in Wistar rats. Life Sci..

[262] Liu J., Wang S., Yi R., Long X., Zhao X. (2022). Effect of Lactobacillus fermentum ZS40 on the NF-κB signaling pathway in an azomethane-dextran sulfate sodium-induced colon cancer mouse model. Front. Microbiol..

[263] Ma F., Song Y., Sun M., Wang A., Jiang S., Mu G., Tuo Y. (2021). Exopolysaccharide Produced by Lactiplantibacillus plantarum-12 Alleviates Intestinal Inflammation and Colon Cancer Symptoms by Modulating the Gut Microbiome and Metabolites of C57BL/6 Mice Treated by Azoxymethane/Dextran Sulfate Sodium Salt. Foods.

[264] Liu M., Xie W., Wan X., Deng T. (2020). Clostridium butyricum modulates gut microbiota and reduces colitis associated colon cancer in mice. Int. Immunopharmacol..

[265] Fahmy C.A., Gamal-Eldeen A.M., El-Hussieny E.A., Raafat B.M., Mehanna N.S., Talaat R.M., Shaaban M.T. (2019). Bifidobacterium longum Suppresses Murine Colorectal Cancer through the Modulation of oncomiRs and Tumor Suppressor miRNAs. Nutr. Cancer.

[266] Talero E., Bolivar S., Ávila-Román J., Alcaide A., Fiorucci S., Motilva V. (2015). Inhibition of chronic ulcerative colitis-associated adenocarcinoma development in mice by VSL#3. Inflamm. Bowel Dis..

[267] Kumar M., Kissoon-Singh V., Coria A.L., Moreau F., Chadee K. (2017). Probiotic mixture VSL#3 reduces colonic inflammation and improves intestinal barrier function in Muc2 mucin-deficient mice. Am. J. Physiol. Gastrointest. Liver Physiol..

[268] Cruz B., Conceição L.L.D., Mendes T.A.O., Ferreira C., Gonçalves R.V., Peluzio M. (2020). Use of the synbiotic VSL#3 and yacon-based concentrate attenuates intestinal damage and reduces the abundance of Candidatus Saccharimonas in a colitis-associated carcinogenesis model. Food Res. Int..

[269] Yu J., Zhou B., Zhang S., Yin H., Sun L., Pu Y., Zhou B., Sun Y., Li X., Fang Y. (2022). Design of a self-driven probiotic-CRISPR/Cas9 nanosystem for sono-immunometabolic cancer therapy. Nat. Commun..

[270] Grenda A., Grenda T., Domaradzki P., Kwiatek K. (2022). Enterococci-Involvement in Pathogenesis and Therapeutic Potential in Cancer Treatment: A Mini-Review. Pathogens.

[271] Bhalla P., Rengaswamy R., Karunagaran D., Suraishkumar G.K., Sahoo S. (2022). Metabolic modeling of host-microbe interactions for therapeutics in colorectal cancer. NPJ Syst. Biol. Appl..

[272] Dong J., Wang B., Xiao Y., Liu J., Wang Q., Xiao H., Jin Y., Liu Z., Chen Z., Li Y. (2024). Roseburia intestinalis sensitizes colorectal cancer to radiotherapy through the butyrate/OR51E1/RALB axis. Cell Rep..

[273] Singh R., Chandrashekharappa S., Bodduluri S.R., Baby B.V., Hegde B., Kotla N.G., Hiwale A.A., Saiyed T., Patel P., Vijay-Kumar M. (2019). Enhancement of the gut barrier integrity by a microbial metabolite through the Nrf2 pathway. Nat. Commun..

[274] Rezaie N., Aghamohammad S., Haj Agha Gholizadeh Khiavi E., Khatami S., Sohrabi A., Rohani M. (2024). The comparative anti-oxidant and anti-inflammatory efficacy of postbiotics and probiotics through Nrf-2 and NF-kB pathways in DSS-induced colitis model. Sci. Rep..

[275] Aboulgheit A., Karbasiafshar C., Zhang Z., Sabra M., Shi G., Tucker A., Sodha N., Abid M.R., Sellke F.W. (2021). Lactobacillus plantarum probiotic induces Nrf2-mediated antioxidant signaling and eNOS expression resulting in improvement of myocardial diastolic function. Am. J. Physiol. Heart Circ. Physiol..

[276] Saeedi B.J., Liu K.H., Owens J.A., Hunter-Chang S., Camacho M.C., Eboka R.U., Chandrasekharan B., Baker N.F., Darby T.M., Robinson B.S. (2020). Gut-Resident Lactobacilli Activate Hepatic Nrf2 and Protect Against Oxidative Liver Injury. Cell Metab..

[277] You T., Zhao Y., Liu S., Xu H. (2023). Lactiplantibacillus plantarum P101 Attenuated Cyclophosphamide-Induced Liver Injury in Mice by Regulating the Nrf2/ARE Signaling Pathway. Int. J. Mol. Sci..

[278] Patra S., Sahu N., Saxena S., Pradhan B., Nayak S.K., Roychowdhury A. (2022). Effects of Probiotics at the Interface of Metabolism and Immunity to Prevent Colorectal Cancer-Associated Gut Inflammation: A Systematic Network and Meta-Analysis With Molecular Docking Studies. Front. Microbiol..

[279] Lingappan K. (2018). NF-κB in Oxidative Stress. Curr. Opin. Toxicol..

[280] Parang B., Barrett C.W., Williams C.S. (2016). AOM/DSS Model of Colitis-Associated Cancer. Methods Mol. Biol..

[281] Do E.J., Hwang S.W., Kim S.Y., Ryu Y.M., Cho E.A., Chung E.J., Park S., Lee H.J., Byeon J.S., Ye B.D. (2016). Suppression of colitis-associated carcinogenesis through modulation of IL-6/STAT3 pathway by balsalazide and VSL#3. J. Gastroenterol. Hepatol..

[282] Liu Y., Yu Z., Zhu L., Ma S., Luo Y., Liang H., Liu Q., Chen J., Guli S., Chen X. (2023). Orchestration of MUC2—The key regulatory target of gut barrier and homeostasis: A review. Int. J. Biol. Macromol..

[283] Iida N., Dzutsev A., Stewart C.A., Smith L., Bouladoux N., Weingarten R.A., Molina D.A., Salcedo R., Back T., Cramer S. (2013). Commensal bacteria control cancer response to therapy by modulating the tumor microenvironment. Science.

[284] Huycke M.M., Abrams V., Moore D.R. (2002). Enterococcus faecalis produces extracellular superoxide and hydrogen peroxide that damages colonic epithelial cell DNA. Carcinogenesis.

